# MANF protein expression is upregulated in immune cells in the ischemic human brain and systemic recombinant MANF delivery in rat ischemic stroke model demonstrates anti-inflammatory effects

**DOI:** 10.1186/s40478-023-01701-y

**Published:** 2024-01-16

**Authors:** Jenni E. Anttila, Olli S. Mattila, Hock-Kean Liew, Kert Mätlik, Eero Mervaala, Päivi Lindholm, Maria Lindahl, Perttu J. Lindsberg, Kuan-Yin Tseng, Mikko Airavaara

**Affiliations:** 1 Drug Research Program, Division of Pharmacology and Pharmacotherapy, Faculty of Pharmacy, University of Helsinki, Viikinkaari 5E, P.O. Box 56, 00014 Helsinki, Finland; 2 Department of Neurological Surgery, Tri-Service General Hospital and National Defense Medical Center, Taipei, 114 Taiwan; 3 Institute of Biotechnology, University of Helsinki, Helsinki, Finland; 4 Department of Neurology, Helsinki University Hospital and Clinical Neurosciences, University of Helsinki, 00290 Helsinki, Finland; 5 Department of Medical Research, Hualien Tzu Chi Hospital, Buddhist Tzu Chi Medical Foundation, Hualien County, Hualien, 970 Taiwan; 6 Department of Pharmacology, Faculty of Medicine, University of Helsinki, Helsinki, Finland; 7 Neuroscience Center, University of Helsinki, 00014 Helsinki, Finland

**Keywords:** Distal middle cerebral artery occlusion, Ischemia, Inflammation, Mesencephalic astrocyte-derived neurotrophic factor, Neuroprotection

## Abstract

Mesencephalic astrocyte-derived neurotrophic factor (MANF) has cytoprotective effects on various injuries, including cerebral ischemia, and it can promote recovery even when delivered intracranially several days after ischemic stroke. In the uninjured rodent brain, MANF protein is expressed almost exclusively in neurons, but post-ischemic MANF expression has not been characterized. We aimed to investigate how endogenous cerebral MANF protein expression evolves in infarcted human brains and rodent ischemic stroke models. During infarct progression, the cerebral MANF expression pattern both in human and rat brains shifted drastically from neurons to expression in inflammatory cells. Intense MANF immunoreactivity took place in phagocytic microglia/macrophages in the ischemic territory, peaking at two weeks post-stroke in human and one-week post-stroke in rat ischemic cortex. Using double immunofluorescence and mice lacking MANF gene and protein from neuronal stem cells, neurons, astrocytes, and oligodendrocytes, we verified that MANF expression was induced in microglia/macrophage cells in the ischemic hemisphere. Embarking on the drastic expression transition towards inflammatory cells and the impact of blood-borne inflammation in stroke, we hypothesized that exogenously delivered MANF protein can modulate tissue recovery processes. In an attempt to enhance recovery, we designed a set of proof-of-concept studies using systemic delivery of recombinant MANF in a rat model of cortical ischemic stroke. Intranasal recombinant MANF treatment decreased infarct volume and reduced the severity of neurological deficits. Intravenous recombinant MANF treatment decreased the levels of pro-inflammatory cytokines and increased the levels of anti-inflammatory cytokine IL-10 in the infarcted cortex one-day post-stroke. In conclusion, MANF protein expression is induced in activated microglia/macrophage cells in infarcted human and rodent brains, and this could implicate MANF’s involvement in the regulation of post-stroke inflammation in patients and experimental animals. Moreover, systemic delivery of recombinant MANF shows promising immunomodulatory effects and therapeutic potential in experimental ischemic stroke.

**Supplementary Information:**

The online version contains supplementary material available at 10.1186/s40478-023-01701-y.

## Introduction

Stroke is one of the leading causes of adult death and disability [16]. Despite this, intravenous thrombolysis remains the only well-established pharmacological treatment available. Inflammation is a major pathophysiological event in the post-stroke brain, initiated by activation of brain resident immune cells, microglia, and infiltration of peripheral leukocytes [30]. Post-stroke inflammation can further exacerbate injury but also serves tissue repair. Thus, more profound knowledge of the mechanisms of ischemic injury and post-ischemic inflammation is still urgently needed to kindle novel therapeutic strategies.

Mesencephalic astrocyte-derived neurotrophic factor (MANF) is an 18 kDa protein widely expressed in different tissues, including the brain, and has cytoprotective properties [31, 39, 41, 45, 54]. In an uninjured brain, MANF protein is expressed mainly in neurons [14, 40] and its expression levels are increased upon acute ischemia [7, 48, 80]. Interestingly, at 24 h after ischemia, MANF protein expression has been reported also in microglia/macrophages of the ischemic region [60, 77], but the protein expression of MANF in later post-ischemic timepoints has not been characterized in animal models of stroke. No previous studies have reported MANF expression in the human stroke brain. We and others have observed that in naïve, non-injured, rodent brains endogenous MANF protein expression is primarily localized in neurons. However, mRNA levels are relatively high in all brain cell types.

Endogenous MANF is localized in the endoplasmic reticulum (ER) lumen but can also be secreted from cells, especially after ER Ca^2+^ depletion [7, 21, 26, 65], and *Manf* gene is induced upon the activation of unfolded protein response of the ER [33, 65]. Intracellular MANF significantly maintains ER protein folding homeostasis and reduces ER stress-induced apoptosis [7, 25, 38]. MANF has been shown to directly interact with several ER luminal proteins, including the chaperone protein GRP78 (a.k.a. BiP), [15, 21, 76] and the ER stress sensors IRE1α, ATF6, and PERK [32]. ER stress sensors are also known to modulate inflammation, and MANF has been shown to affect inflammation processes. Endogenous neuronal MANF protects against cerebral ischemia since embryonic *Manf* deletion from neuronal lineage cells leads to larger infarcts in *Nestin*^*Cre/*+^*::Manf*^*flox/flox(fl/fl)*^ mice compared to wild type mice [48].

Exogenous MANF protects brain tissue from ischemic damage in rat models of ischemic stroke when delivered intracranially as a protein or via viral vector [2, 3, 23, 50, 72, 79] and, more importantly, alleviates functional deficits when injected intracranially several days after cerebral ischemia [6, 48]. The mode of action of exogenous MANF remains unclear, but immunomodulatory effects have been suggested as a putative mechanism for MANF’s cytoprotective properties. Administration of recombinant MANF into mouse brain has been shown to downregulate inflammation by decreasing NF-κB-mediated pro-inflammatory cytokine production in vivo in an ischemic stroke model in aged mice [23] and in vitro after oxygen–glucose deprivation [23, 83].

We have previously shown that viral-vector mediated overexpression of MANF in the peri-infarct region increases the number of phagocytic microglia/macrophages after ischemic stroke [48] and downregulates proteins S100A8 and S100A9 related to innate immunity [66]. Furthermore, MANF has been shown to increase the pro-regenerative and anti-inflammatory activation, known as alternative activation, of innate immune cells in an ischemic stroke model [77] and the damaged retina of mouse and fruit fly [51]. However, MANF does not penetrate the blood–brain barrier. So far, MANF therapy has been administered by intracranial delivery, which is highly invasive and thus not a realistic approach for therapeutic use. Intranasal delivery of several other proteins is neuroprotective in rat and mouse transient middle cerebral artery occlusion models [12, 22, 43, 78]. In principle, intranasally delivered molecules can bypass the blood–brain barrier (BBB) and access the central nervous system via several pathways, including the olfactory and trigeminal nerves, vascular and cerebrospinal fluid pathways, and the lymphatic and glymphatic systems [24, 44, 67].

Since the knowledge of post-stroke MANF protein expression after cerebral ischemia is limited to acute time-points (24–48 h) and data from human patients is lacking, the initial aim of our study was to characterize the evolution of endogenous MANF protein expression in the ischemic human brain. The emphasis on human stroke brain samples is crucial as it not only provides a clinically relevant perspective but allows identifying potential species-specific differences in MANF protein expression, which need to be taken into account for translational research and therapeutic development. We then studied whether we observe a similar expression pattern in rodent models of ischemic stroke, and identified which cells express MANF protein. Secondly, we conducted a proof-of-concept study using non-invasive intranasal delivery of recombinant human MANF (rhMANF) for neuroprotection in a rat model of distal middle cerebral artery occlusion (dMCAo) model. The findings from our intranasal rhMANF study led us to the third aim: demonstrate the effects of intravenously administered rhMANF in the dMCAo model.

We show for the first time how MANF protein expression evolves temporally in the post-stroke human brain and similarly in the rat brain. We demonstrate that after stroke, activated microglia/macrophages prominently express MANF in both species and that stroke causes a clear transition of MANF protein expression pattern towards microglia/macrophages. Moreover, we show that systemic delivery of rhMANF reduces ischemic cerebral injury. The protective effect can be mediated via the downregulation of pro-inflammatory cytokine and upregulation of anti-inflammatory cytokine production in the infarcted cortex. These data strengthen the translational relevance of MANF as an endogenous cytoprotective mediator, both in neurons and in phagocytic microglia/macrophage cells post-stroke. Systemic administration of MANF presents a novel therapeutic approach to utilize these cytoprotective and anti-inflammatory properties in ischemic stroke.

## Materials and methods

### Patients

We studied MANF immunoreactivity in 7 acute ischemic stroke patients treated at our hospital and who had died early after stroke onset. The rapid autopsy procedures and sample collection have been described in detail previously [42, 56, 57]. The study had the approval of the local research ethics committee, and next of kin gave informed consent for the study. Patient details are given in Table 2.

### Immunohistochemistry on human brain sections

On autopsy, 1 cm^3^ cortical samples including white matter were dissected, formaldehyde fixed and embedded in paraffin. Tissue sampling sites including infarcted brain tissue and contralateral healthy tissue were decided based on the individual topography of each brain infarction, using macroscopic examination of the parenchyma and cerebrovasculature, and comparing it to the most recent computed tomography scans [42, 56, 57]. Briefly, on autopsy, the infarcted brain areas were identified during the macroscopic examination of the brain parenchyma and cerebrovasculature in comparison with the most recent computed tomography scans. Since the localization and size of the infarcts were unique in each case, we preferred to target the tissue sampling on the basis of the individual infarct topography rather than standard localizations. Samples from the corresponding areas of the contralateral or non-infarcted hemispheres were processed in a similar way. Examination of the hematoxylin–eosin–stained sections to grade the severity of ischemic neuronal changes was performed by a neuropathologist without information of the sample localization. Focusing on the integrity of the nucleus, we ascribed scores for signs of ischemic neuronal changes to each tissue section as follows: 1, largely normal morphology but scattered neurons had nuclear abnormalities such as pyknosis, low nuclear cytoplasmic contrast, or smearing of nuclear border (similar to type III neurons); 2, a large proportion of neurons had nuclear abnormalities; 3, a large proportion of neurons had nuclear abnormalities while scattered ones exhibited signs of irreversible damage such as shrunken cytoplasm with irregular borders and invisible nuclei (similar to type IV neurons); and 4, a large proportion of neurons showed irreversible changes. These characteristic morphological neuronal changes are well appreciated in light microscopy after > 24 h following the ischemia onset in large, fatal infarctions. The ischemic neuronal changes were scored in each sample with a focus on the integrity of the nucleus (Table 2).

The formalin-fixed, paraffin-embedded samples were cut into 4 µm sections and mounted on microscope slides. The sections were deparaffinized and heated in 10 mM citrate buffer, pH 6.0 for antigen retrieval. For anti-MANF immunostaining, endogenous peroxidase activity was blocked with 0.3% hydrogen peroxide in methanol (Sigma Aldrich), and the non-specific antibody binding was blocked with 10% normal goat serum (cat#PK-6101, Vector Laboratories, Burlingame, CA, USA) in 0.1% Tween-20 (Sigma Aldrich) in TBS (TBS-T). To further reduce non-specific staining, the sections were blocked with avidin and biotin (Vector Laboratories, cat# SP-2001) followed by incubation with rabbit anti-MANF (1:800, cat#HPA011175, Atlas antibodies, Bromma, Sweden) in 1.5% normal goat serum in 0.1% TBS-T at 4 °C overnight. The next day, sections were incubated with secondary antibody (biotinylated goat anti-rabbit 1:200, cat#PK-6101, Vector Laboratories) followed by incubation with avidin–biotin complex (ABC kit, cat#PK-6101, Vector Laboratories). The color was developed using a peroxidase reaction with 3´,3´-diaminobenzidine (DAB; cat#SK-4100, Vector Laboratories), the sections were counterstained with hematoxylin, dehydrated, and coverslipped. Rabbit IgG (cat#NI01, Merck Millipore, Temecula, CA, USA) was used as a negative control instead of the primary antibody using the same protein concentration as with the primary antibody. For anti-CD68 staining, the Novolink™ Polymer Detection System kit (cat#RE7140-K, Leica Biosystems, Newcastle Upon Tyne, UK) was used according to manufacturer’s instructions. Briefly, after antigen retrieval, the sections were incubated for 5 min with peroxidase block, washed, incubated for 5 min with protein block, washed, and incubated for 1 h at room temperature with mouse anti-human CD68 (1:200, clone PG-M1, cat#M0876, Dako, Glostrup, Denmark). After washing, the sections were incubated for 30 min with post primary, washed, incubated for 30 min with Novolink™ polymer, washed, and incubated with DAB chromogen. Mouse IgG_3_ isotype control (cat#MAB007, R&D Systems, Minneapolis, MN, USA) was used as a negative control instead of the primary antibody using the same protein concentration as with the primary antibody.

The samples were scanned with a 3DHISTECH Pannoramic 250 FLASH II digital slide scanner (scanning service provided by the Institute of Biotechnology, University of Helsinki; https://www.helsinki.fi/en/infrastructures/histotechnology-and-laboratory-animal-pathology/bi-histoscanner) and the images of MANF and CD68 immunostainings from each patient sample were taken from the same location of section with the Pannoramic Viewer programme, version 1.15.3.

### Animals

All the animal experiments comply with current Finnish and Taiwanese laws.

*Finland* A total of 140 male Sprague Dawley rats (age 7–8 weeks, weight 200–270 g, Envigo, Netherlands) and 32 C57BL/6NHsd male mice (age 8–9 weeks, weight 22–28 g, Envigo, Netherlands) were used for the experiments. Gene-modified *Nestin*^*Cre/*+^::*Manf*^*fl/fl*^ male mice (n = 4) were used to investigate post-stroke MANF expression after MANF deletion from neuronal lineage cells. *Manf*^*fl/fl*^ male littermates (n = 5) were used as controls. The generation of *Manf*^*fl/fl*^ and *Nestin*^*Cre/*+^::*Manf*^*fl/fl*^ mice has been described in detail before [38]. *Manf* exon 3 was conditionally removed by crossing the *Manf*^*fl/fl*^ mice with Nestin-Cre transgenic mice (B6.Cg-Tg[Nes-cre]1Kln/J, a gift from Edgar Kramer). Congenic *Manf*^*fl/fl*^ or *Manf*^*fl/*+^ mice in C57BL/6JRcc background were crossed with either *Nestin*^*Cre/*+^*::Manf*^*fl/fl*^ or *Nestin*^*Cre/*+^*::Manf*^*fl/*+^ mice to generate *Nestin*^*Cre/*+^*::Manf*^*fl/fl*^ and *Manf*^*fl/fl*^ mice. All animal experiments were conducted according to the 3R principles of EU directive 2010/63/EU on the care and use of experimental animals, and according to local laws and regulations, and were approved by the national Animal Experiment Board of Finland (protocol approval number ESAVI/5459/04.10.03/2011 and ESAVI/7812/04.10.07/2015). The animals were housed in groups of 4–5 with ad libitum access to food and water under a 12 h/12 h dark–light cycle and the well-being of the animals was monitored daily.

*Taiwan* A total of 42 male Sprague–Dawley rats (age 12 weeks; weight 250–300 g, BioLASCO Taiwan Co., Ltd) were used for the experiments. Animals were housed in groups of 3–4 rats under a 12-h light/dark cycle at a controlled temperature (22 °C) and humidity (50%) with free access to food and water. The animal experiments were carried out in accordance with the National Institute of Health Guide for the Care and Use of Laboratory Animals. Experimental protocols were approved by the Institutional Animal Care and Use Committee of the National Defense Medical Center (approval number IACUC 16258), and Approval of Animal Use Protocol Board Buddhist Tzu Chi General Hospital (approval number IACUC 107-04), Taiwan, R.O.C.

All the experiments were performed in a blinded manner and are reported according to the ARRIVE guidelines. Blinding procedures were such that the person performing the stroke surgery, behavioral testing, and data analysis did not know treatment allocation.

### Distal middle cerebral artery occlusion

*Finland* For rats, cortical cerebral ischemia was induced by occluding the distal middle cerebral artery (dMCA) together with a bilateral common carotid artery (CCA) occlusion [13]. The rats were anesthetized with 4% chloral hydrate (Sigma Aldrich) intraperitoneally (i.p.; 400 mg/kg) and lidocaine (Orion Pharma, Espoo, Finland) was used as a local anesthetic. The surgery was performed as described previously [2, 3, 5, 48]. Briefly, the CCAs were isolated through a cervical incision. A small craniotomy was made on the right side of the skull and the right dMCA was ligated directly with a 10-0 suture. CCAs were simultaneously occluded with non-traumatic arterial clips. After 60 or 90 min, the dMCA and CCAs were reopened to allow reperfusion. For mice, a permanent dMCAo was performed similarly by ligating the dMCA permanently with the 10–0 suture, but without occluding the CCAs. The body temperature of the animals was maintained at 37°C throughout the procedures until recovery from anesthesia when the animals were returned to their home cages. The animals received one dose of carprofen (Rimadyl Vet 50 mg/ml, Zoetis Animal Health, Copenhagen, Denmark) subcutaenously (5 mg/kg) for post-operative pain. A total of 12 rats (6 in the vehicle group, 6 in the rhMANF group) and 3 mice did not survive the surgery. There was no further mortality.

*Taiwan* The procedures were the same as in Finland, except before the reopening of the dMCA, the rats were anesthetized with 5% isoflurane in 30% O_2_ / 70% N_2_O using the V-10 Anesthesia system (VetEquip, Inc., Pleasanton, CA). Following induction of anesthesia, the level of isoflurane was maintained at 1.5% during the procedure. After dMCAo surgery, ketoprofen was administered once at a dose of 2.5 mg/kg per rat for pain relief.

### Intranasal administration

The rats were assigned to different treatment groups in a random manner. RhMANF (P-101-100, Icosagen, Estonia) in phosphate-buffered saline (PBS) or only PBS was administered to the nasal cavity of the rats as described previously [5, 46]. Briefly, the rats were anesthetized with isoflurane (4.5%) or, when administered during dMCAo surgery, 4% chloral hydrate, and 10 µl of rhMANF or PBS was pipetted into each nostril. To test the neuroprotective effect of intranasal rhMANF delivery, rhMANF or PBS was administered at three different time points: 12 h before dMCAo, immediately before dMCAo, and immediately after reperfusion, equaling a total of 20 µg or 60 µg of rhMANF. There was no significant difference between the doses in infarct volume (*p* = 0.49, Student’s *t*-test). Therefore, the 20 µg and 60 µg groups were combined for statistical analysis when compared to the vehicle group.

### Intravenous administration

The rats were assigned to different treatment groups in a random manner. RhMANF in 0.9% NaCl or 0.9% NaCl (vehicle control) were given as an intravenous bolus (500 µl per dose). Either one dose of 1.5 µg rhMANF was injected into the tail vein approximately 15 min after dMCAo reperfusion or three doses of 1.5 µg rhMANF were injected into the femoral vein with 10 min intervals starting 10 min after the dMCAo reperfusion.

### Behavioral tests

The body asymmetry test, modified Bederson’s neurological test and analysis of locomotor activity were assessed in rats as described previously [2, 3, 5, 48]. Briefly, body asymmetry was analyzed from 20 consecutive trials by lifting the rats above the testing table by the tails and counting the frequency of initial turnings of the head or upper body contralateral to the ischemic side (the maximum impairment in stroke animals is 20 contralateral turns whereas naïve animals turn in each direction with equal frequency resulting in 10 contralateral turns) [11]. In the modified Bederson’s score the neurological deficits were scored according to the following criteria: 0 = no observable deficit; 1 point = rats show decreased resistance to lateral push; 2 points = rats keep the contralateral forelimb to the breast and extend the other forelimb straight when lifted by the tail in addition to behavior in score 1; 3 points = rats twist the upper half of their body towards the contralateral side when lifted by the tail in addition to behavior in other scores [9]. Locomotor activity was measured using an infrared activity monitor for one hour (Med Associates, St. Albans, VT, USA).

### Laser Doppler flowmetry

The effect of intranasal rhMANF (10 µg) on cerebral blood flow (CBF) was measured with Laser Doppler flowmetry (LDF; moorVMS-LDF, Moor Instruments, Axminster, UK) during and after dMCAo (n = 19). The skull was thinned by drilling a small hole on the cortex [A/P  − 2.0; L/M 4.0 relative to bregma [53]] and the MoorVMS-LDF1 optical fibre probe was attached on the skull with a PH-DO single fibre holder (Moor Instruments) and dental cement. The baseline CBF was monitored for 10 min before the occlusion of CCAs and dMCA, and monitoring was continued for 10 min after reperfusion. Body temperature was maintained at 37°C with an automated heating pad. One animal was excluded due to insufficient (< 65%) reduction of CBF after stroke induction.

### Blood pressure measurement

The rats (n = 13) were anesthetized with 4% chloral hydrate (400 mg/kg i.p.) and lidocaine (Orion Pharma) was used as a local anesthetic. The trachea was intubated and the measurement probe was inserted into the left common carotid artery. The femoral vein was cannulated for rhMANF or saline administration. The mean arterial blood pressure and heart rate were recorded using PowerLab 8/30 Channel Recorder (ADInstruments) for 40 min. Baseline blood pressure was measured for 10 min and a 500 µl bolus of increasing rhMANF dose (1.5 µg, 15 µg and 150 µg) or saline was injected every 10 min into the femoral vein. The body temperature was maintained at 37°C during all procedures. The animals were euthanized immediately after the measurement.

### Analysis of blood gases and electrolytes

Under chloral hydrate (4%, 400 mg/kg i.p.) anesthesia, the femoral artery was cannulated with a PE-50 polyethylene tube for monitoring of blood gasses and electrolytes (n = 15). Body temperature was automatically maintained at 37.5 ± 0.5°C by a rectal temperature sensor and a heating pad (CMA-150, Sweden). A 100 µl arterial blood sample was withdrawn from the femoral artery and immediately injected into the epoc® Blood Analysis System (Epocal) for analysis of the blood gasses and other physiological parameters including pH, partial pressure of oxygen (pO2), partial pressure of carbon dioxide (pCO2), sodium, potassium, glucose, lactate, and hemoglobin 10 min before the dMCAo (baseline), at dMCAo reperfusion, and at 30 min and 2 h after the reperfusion.

### Immunohistochemistry on rat and mouse brain sections

The animals were deeply anesthetized with pentobarbital (90 mg/kg i.p., Mebunat, Orion Pharma) and transcardially perfused with saline followed by 4% paraformaldehyde. Brains were post-fixed in 4% paraformaldehyde for 2 days, dehydrated in a series of ethanol and xylene, and embedded in paraffin. Brains were cut into 5 µm sections using a Leica HM355S microtome and mounted on Labsolute microscope slides (Th. Geyer, Renningen, Germany).

For chromogenic anti-MANF (1:800) immunostaining, the same protocol was used as for human sections, except with 1.5% goat serum for blocking, and no avidin–biotin blocking was used. Double immunofluorescence stainings with the rabbit anti-MANF antibody (1:200) were performed with goat anti-Iba1 (1:250, cat#ab5076, Abcam, Cambridge, UK), mouse anti-CD68 (1:500, cat#MCA341R, AbD Serotec, Kidlington, UK), and mouse anti-NeuN (1:200, cat#MAB377, Millipore). Goat anti-rabbit Alexa488 (cat#A11034, Life Technologies, Paisley, UK) and goat anti-mouse Alexa568 (cat#A11004, Life Technologies) or donkey anti-rabbit Alexa488 (cat#A21206, Life Technologies) and donkey anti-goat Alexa568 (cat#A11057, Life Technologies) were used as secondary antibodies (1:500), the slides were coverslipped with Vectashield Hardset Antifade Mounting Medium with DAPI (cat#H-1500, Vector Laboratories) and imaged with a Zeiss LSM 700 confocal microscope.

The specificity of the anti-MANF antibody was confirmed with additional pre-adsorption controls and *Nestin*^*Cre/*+^*::Manf*^*fl/fl*^ knockout tissue.

### Analysis of infarction size

The rats were euthanized 2 days after dMCAo, the brains were sliced into seven 2 mm coronal sections, and stained with 2% 2,3,5-triphenyltetrazolium chloride (Sigma Aldrich) in PBS for 15 min at room temperature. The sections were transferred into 4% paraformaldehyde (Sigma Aldrich) for fixation, scanned and analyzed with open-source ImageJ software. To calculate the infarction volume, the infarct area of each section was first corrected for brain swelling by subtracting the area of non-infarcted ipsilateral hemisphere from the total area of contralateral hemisphere [37], then multiplied by the thickness of the section, and finally the infarct volume of each section was summed up to provide a total infarct volume for each animal.

In *Nestin*^*Cre/*+^*::Manf*^*fl/fl*^ knockout mice and *Manf*^*fl/fl*^ control mice, the infarction area was analyzed from hematoxylin stained paraffin sections 14 days post-stroke. The infarction area was delineated in Pannoramic Viewer programme and the infarction size was calculated as percentage of the total brain area of each section. The average infarction size was calculated from three sections per each animal (2 striatal and 1 thalamic section).

### Cytokine ELISA

The ipsilateral cortical tissues and serum were collected 1 day after dMCAo from rats. The tissue samples were homogenized in lysis buffer (PRO-PREPTM, iNtRON Biotechnology, Korea), centrifuged at 12 000 g for 30 min, and the supernatants were collected and stored at -80 °C. The cytokine (TNFα, IL-1β, and IL-6, IL-10) levels were quantified using commercial ELISA kits (DY510; DY501; DY506; DY522; R&D Systems Minneapolis, MN, USA) according to manufacturer's instructions. The cytokine levels were normalized to the total protein concentration of the sample.

### Mouse MANF ELISA

Endogenous MANF levels were quantified from mouse serum after permanent dMCAo with an in-house double antibody sandwich mouse MANF (mMANF) ELISA. Terminal blood samples were taken by cardiac puncture 1 h, 6 h, 24 h and 48 h after permanent dMCAo. The blood was let to coagulate for at least 30 min and serum was separated by centrifugation with 2,000 g at room temperature for 10 min and stored at −80°C until analysis. Sera were diluted 1:40 for quantitation. The development and validation of mMANF ELISA were described in detail elsewhere [20]. The dynamic range of mMANF ELISA is 31.25–1000 pg/ml, and sensitivity 29 pg/ml. The assay detects both mouse and human MANF and does not give a signal from MANF knockout tissues. In brief, 96-well MaxiSorp plates were coated with goat anti-human MANF antibody (AF3748, R&D Systems) in 50 mM carbonate buffer, pH 9.6. After blocking with 1% casein-PBST (PBS; 0.05% Tween 20), mouse sera and standard samples (mMANF; CYT-827, ProSpec, Rehovot, Israel) diluted in blocking buffer were applied to the plate and incubated overnight at +4°C. After washing, bound MANF in the wells was detected using rabbit anti-MANF antibody (LS-B2688, LSBio, Seattle, WA, USA), followed by incubation with horseradish peroxidase (HRP)-conjugated anti-rabbit IgG secondary antibody (NA9340V, GE Healthcare, Chicago, IL, USA). The color signal was developed using the DuoSet ELISA Development System and absorbance was read at 450 nm and 540 nm using a VICTOR3 plate reader (PerkinElmer, Waltham, MA, USA).

### ^***125***^***I-labeled MANF***

The rats (n = 10) underwent 60 min dMCAo together with CCA occlusion. Immediately after reperfusion, a mixture of unlabeled rhMANF (1 µg/µl, Icosagen) and ^125^I-rhMANF (approximately 1.4 ng/µl; 54058 CPM/µl), labeled by lactoperoxidase*-*catalyzed radioiodination [10], in 80 mM Na-phosphate buffer (pH 7.5) containing 1% bovine serum albumin (Sigma Aldrich), was administered intranasally (10 µl per each nostril). After 60 min the animals were perfused transcardially with 200 ml of saline. Blood samples were collected by cardiac puncture before perfusion. Liver samples were collected after perfusion and used for radiolabel quantification without homogenization. The whole brains were collected and homogenized in ELISA lysis buffer as described below. Half of the brain lysate was used for measuring the amount of radioactivity (counts per minute) and the signal was normalized to the original brain weight. Radioiodine content was quantified using the Perkin Elmer-Wallac Wizard 1480 Gamma Counter. Background radioactivity was subtracted from the gamma counts.

### Sample processing for hMANF ELISA

Blood samples were taken either from the tail vein or by cardiac puncture before perfusion. Sera were prepared as described above and stored at −80°C until analysis. Before the brains were collected, the animals were perfused transcardially with 200 ml of saline to remove blood. The whole brain was snap-frozen in isopentane on dry ice and stored in −80°C until homogenization. The whole brains of rats treated with intranasal ^125^I-labeled rhMANF were homogenized by grinding in liquid nitrogen and lysed with ELISA lysis buffer (137 mM NaCl; 20 mM Tris–HCl, pH 8.0; 2.5 mM EDTA; 1% NP40; 10% glycerol; 660 mg tissue/ml) containing protease inhibitors (Complete, Mini, EDTA-free Protease Inhibitor Cocktail, Roche, Mannheim, Germany). Half of the brain lysate was further processed for hMANF ELISA and half of the lysate was used as such to measure gamma counts as described above. From the brains of rats treated with i.v. rhMANF, the infarcted cortex and corresponding contralateral cortex were dissected out and homogenized with ELISA lysis buffer containing protease inhibitors. The brain lysates were incubated on ice for at least 20 min, centrifuged 15 000 g at 4°C for 20 min, and the supernatants were collected.

### Human MANF ELISA

The rhMANF protein levels from rat brain supernatants and serum were analyzed with an in-house double antibody sandwich ELISA specific for human MANF (hMANF) as described previously except without heterophilic antibody blocker [18]. The dynamic range of hMANF ELISA is 62.5–2000 pg/ml, and sensitivity 45 pg/ml. The sera samples were diluted at 1:10, 1:20, 1:75 or 1:100 and brain homogenates at 1:2 in blocking buffer [1% casein in PBS; 0.05% Tween 20 (PBST)] for quantitation. Briefly, 96-well MaxiSorp (Nunc, Fischer Scientific, Waltham, MA, USA) were coated with goat anti-human MANF antibody (AF3748, R&D Systems, Minneapolis, MN, USA) in 50 mM carbonate buffer, pH 9.6. Wells were blocked using blocking buffer, and diluted samples and standards (hMANF; P-101-100, Icosagen) were applied to wells for duplicate measurements, and incubated overnight at +4°C. After washing with PBST, HRP-conjugated mouse anti-human MANF antibody (4E12, Icosagen) was applied to the wells and incubated for 5 h at room temperature. HRP signal was generated using DuoSet ELISA Development System (R&D Systems) according to manufacturer’s instructions, and the absorbance was read at 450 nm and 540 nm (for wavelength correction) using a VICTOR3 plate reader (Perkin Elmer). The rhMANF concentration in brain supernatant was normalized to the total protein concentration of the sample determined with the Lowry method (DC Protein Assay, Bio-rad Laboratories, Hercules, CA, USA).

### Evans blue extravasation

The integrity of blood–brain barrier (BBB) was evaluated with Evans blue extravasation 2 days after dMCAo [36]. The rats (n = 13) were anesthetized with sodium pentobarbital (50 mg/kg, i.p.) and infused via the right femoral vein with 37°C Evans blue dye (2% in 0.9% normal saline, 4 ml/kg) over 5 min. Two hours later, the rats were perfused with 300 ml of normal saline to wash out any remaining dye in the blood vessels and then the brains were removed and sectioned to 2 mm thickness with a rodent brain matrix. Coronal brain sections were taken starting at + 2 mm and ending at − 2 mm from bregma. BBB permeability was evaluated in the contralateral non-ischemic cortex, ipsilateral ischemic cortex, and in the cerebellum. The cerebellum was used as an internal control. Each tissue sample was weighed immediately and placed in 0.5 ml of 0.9% normal saline for homogenization of the sample. For protein precipitation, 0.5 ml of 60% trichloroacetic acid solution was added and vortexed for 2 min. The mixture was subsequently cooled down at 4°C for 30 min and centrifuged (1500 g at 4°C) for another 30 min. The absorbance of Evans blue in the supernatant was then measured with a spectrophotometer (Molecular Devices OptiMax, USA) at 610 nm. The amount of Evans blue dye in the sample was calculated from a standard curve obtained from known amounts of the dye and was expressed as μg/g of the net tissue weight.

### Statistical analysis

GraphPad Prism (version 9.2.0, GraphPad Software, San Diego, CA, USA) was used for statistical analysis. Normal distribution of each dataset was analyzed by Shapiro–Wilk test. Normally distributed data were analyzed with two-tailed Student’s *t*-test, one-way ANOVA or two-way repeated-measures ANOVA. Data with non-normal distribution were analyzed with nonparametric Mann–Whitney U test and corrected for multiple comparisons with the Holm-Šídák method, when applicable. The multiple comparisons adjusted *p* values are reported for the Mann-Whitney U test. Statistical significance was considered at *p* < 0.05. The results are presented as mean ± standard deviation. All the used statistical tests, related to each figure, and their results are shown in the statistical table (Table 1). Exclusion criteria: In order to validate the effectiveness of the middle cerebral artery occlusion (MCAo) procedure, we meticulously assessed the accuracy of ligation. As exclusion criteria, animal that exhibited evidence of unsuccessful ligation resulting in an absence of stroke-induced lesions was eliminated from the study, and this was determined by the laser Doppler measurements.

**Table 1:** Statistical table

	Dataset	Data structure	Type of test	Power
a	Figure 4i	Normal distribution (Shapiro–Wilk *p* = 0.115)	t-test	*p* = 0.641
b	Figure 4n	Normal distribution (Shapiro–Wilk *p* = 0.942)	One-way ANOVA	F(4,18) = 0.41; *p* = 0.801
c	Figure 5b	Normal distribution (Shapiro–Wilk *p* = 0.222)	t-test	*p* = 0.038
d	Figure 5c	Non-normal distribution (Shapiro–Wilk *p* < 0.0001)	Mann–Whitney U	Adjusted *p* values: Section 1: *p* = 0.900 Section 2: *p* = 0.900 Section 3: *p* = 0.664 Section 4: *p* = 0.195 Section 5: *p* = 0.121 Section 6: *p* = 0.201 Section 7: *p* = 0.900
e	Figure 5e	Non-Normal distribution (Shapiro–Wilk *p* = 0.016)	Mann–Whitney U	Adjusted *p* values: *p* ≥ 0.771
f	Figure 5f	Non-normal distribution (Shapiro–Wilk *p* < 0.0001)	Mann–Whitney U	Adjusted *p* values: d2: *p* = 0.750 d7: *p* = 0.051 d14: *p* = 0.0004
g	Figure 5g	Non-normal distribution (Shapiro–Wilk *p* < 0.0001)	Mann–Whitney U	Adjusted *p* values: d2: *p* = 0.715 d7: *p* = 0.058 d14: *p* = 0.0003
h	Figure 5h	Normal distribution (Shapiro–Wilk *p* = 0.109)	Two-way RM ANOVA	Time x Treatment interaction: F(2,54) = 0.13; *p* = 0.878
i	Figure 5i	Normal distribution (Shapiro–Wilk *p* = 0.172)	Two-way RM ANOVA	Time x Treatment interaction: F(2,54) = 0.01; *p* = 0.993
j	Figure 5j	Normal distribution (Shapiro–Wilk *p* = 0.241)	Two-way RM ANOVA	Time x Treatment interaction: F(3,81) = 0.39; *p* = 0.760
k	Figure 6b	Normal distribution (Shapiro–Wilk *p* = 0.674)	t-test	*p* = 0.006
l	Figure 6c	Non-normal distribution (Shapiro–Wilk *p* < 0.0001)	Mann–Whitney	Adjusted *p* values: Section 1: *p* = 0.244 Section 2: *p* = 0.244 Section 3: *p* = 0.209 Section 4: *p* = 0.209 Section 5: *p* = 0.113 Section 6: *p* = 0.209 Section 7: *p* = 0.244
m	Figure 7b	Normal distribution (Shapiro–Wilk *p* = 0.833)	t-test	*p* < 0.0001
n	Figure 7c	Normal distribution (Shapiro–Wilk *p* = 0.908)	t-test	*p* < 0.0001
o	Figure 7d	Normal distribution (Shapiro–Wilk *p* = 0.900)	t-test	*p* < 0.0001
p	Figure 7e	Normal distribution (Shapiro–Wilk *p* = 0.167)	t-test	*p* < 0.0001
q	Figure 7f	Non-Normal distribution (Shapiro–Wilk *p* = 0.025)	Mann–Whitney U	*p* = 0.005
r	Figure 7g	Non-Normal distribution (Shapiro–Wilk *p* = 0.032)	Mann–Whitney U	*p* = 0.511
s	S Fig. 2c	Normal distribution (Shapiro–Wilk *p* = 0.480)	t-test	*p* = 0.038
t	S Fig. 3b	Non-Normal distribution (Shapiro–Wilk *p* = 0.007)	Mann–Whitney U	*p* = 0.015
u	S Fig. 3c	Non-Normal distribution (Shapiro–Wilk *p* < 0.0001)	Mann–Whitney U	Adjusted *p* values: 30 min: *p* = 0.004 1 h: *p* = 0.004
v	S Fig. 4b	Non-Normal distribution (Shapiro–Wilk *p* = 0.004)	Mann–Whitney U	Adjusted *p* values: *p* ≥ 0.111
w	S Fig. 4c	Normal distribution (Shapiro–Wilk *p* = 0.099)	Two-way RM ANOVA (Mixed-effects model)	Time x Treatment interaction: F(3,38) = 2.66; *p* = 0.062
x	S Fig. 4d	Non-Normal distribution (Shapiro–Wilk *p* = 0.044)	Mann–Whitney U	Adjusted *p* values: *p* ≥ 0.151
y	S Fig. 4e	Non-Normal distribution (Shapiro–Wilk *p* = 0.018)	Mann–Whitney U	Adjusted *p* values: *p* ≥ 0.733
z	S Fig. 4f	Non-Normal distribution (Shapiro–Wilk *p* = 0.012)	Mann–Whitney U	Adjusted *p* values: *p* ≥ 0.340
aa	S Fig. 4g	Non-Normal distribution (Shapiro–Wilk *p* = 0.0005)	Mann–Whitney U	Adjusted *p* values: *p* ≥ 0.419
ab	S Fig. 4h	Normal distribution (Shapiro–Wilk *p* = 0.159)	Two-way RM ANOVA (Mixed-effects model)	Time x Treatment interaction: F(3,38) = 0.70; *p* = 0.555
ac	S Fig. 4i	Non-Normal distribution (Shapiro–Wilk *p* = 0.0006)	Mann–Whitney U	Adjusted *p* values: *p* ≥ 0.186
ad	S Fig. 4k	Non-Normal distribution (Shapiro–Wilk *p* < 0.0001)	Mann–Whitney U	Adjusted *p* values: *p* ≥ 0.999
ae	S Fig. 4l	Non-Normal distribution (Shapiro–Wilk *p* < 0.0001)	Mann–Whitney U	Adjusted *p* values: *p* ≥ 0.997
af	S Fig. 5b	Normal distribution (Shapiro–Wilk *p* = 0.715)	t-test	*p* = 0.093

*RM* Repeated measures

## Results

### Ischemic stroke induces delayed MANF protein expression in brain monocyte lineage cells in humans

**Fig. 1:**
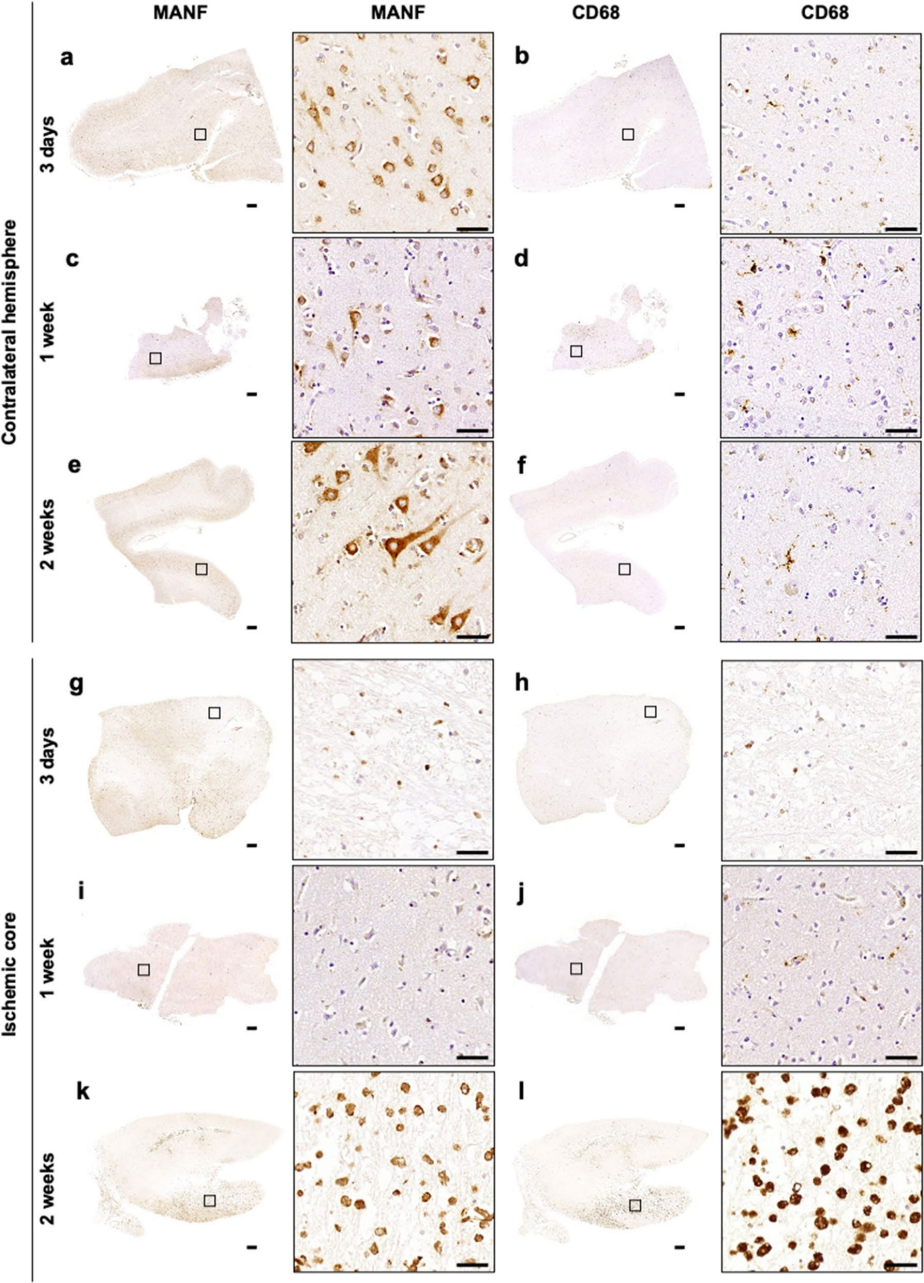
MANF protein expression correlates with CD68 expression at 2 weeks after ischemic stroke in the human brain. Anti-MANF and anti-CD68 immunostaining of human cerebral tissue from ischemic stroke patients at 3d, 1wk and 2wk post-stroke. The patient and sample details are shown in Table 2. **a-f** Contralateral hemisphere of the ischemic brain (**a**, **b** Patient 1, frontal region; **c**, **d** Patient 4, frontal region; **e**, **f** Patient 7, parietal region) **g-l** The ischemic core (**g**, **h** Patient 1, pons; **i**, **j** Patient 4, frontal region; **k**, **l** Patient 7, parietal region. Scale bar is 1000 µm and 50 µm. Location of high magnification images indicated in the whole section images

To characterize MANF protein expression in the infarcted human brain, we utilized immunohistochemistry for MANF and CD68 in consecutive *postmortem* tissue sections of patients deceased approximately 3 days, 1 week and 2 weeks after ischemic stroke (n = 2–3 per time point; Table 2). Samples from the contralateral non-infarcted hemisphere were used for comparison. In the contralateral hemisphere, MANF expression was mainly neuronal at all time points studied (Fig. 1a, c, e). On the contrary, in the infarct core we did not observe MANF expression in neurons between 3 days and 2 weeks post-stroke. In fact, limited MANF immunoreactivity was observed at day 3 and at 1-week post-stroke. MANF expression in the ischemic area of 1-week post-stroke samples was very low. However, in the 2 weeks post-stroke samples, high numbers of MANF and CD68 positive round cells were detected in the infarct region (Fig. 1k, l). Overall, the number and location of MANF and CD68 positive cells correlated in all but one of the ipsilateral hemisphere samples (Table 2). Less correlation between MANF and CD68 expressing cells was observed in the contralateral side due to the neuronal expression of MANF. However, we observed CD68 positive cells also in the contralateral side, especially in the white matter. Some of these white matter cells were also MANF positive but fewer than in the ipsilateral hemisphere. The morphology of CD68 positive cells in the contralateral hemisphere was more ramified than in the infarcted hemisphere.

**Table 2:** Characteristics of the ischemic stroke patient samples and MANF and CD68 immunoreactivity in ipsilateral (ipsi) and contralateral (contra) hemispheres

Pa-tient no	Survival time	Occluded vessel^a^ HT^b^	Cause of death^c^ Agonal phase^*^	Age / Gender	Risk factors^d^	Hemi-sphere	Sampled brain region^e^	Neuro-score^f^	Immunoreactivity^g^		
									Neuronal MANF	Non-neuronal MANF	CD68
1	2.5 d	BA/T, HT	Stroke^*^	74 / M	AS, CAD	Ipsi	C	2	0	+ + +	+ + +
						Ipsi	P	3	+	+ +	+ +
						Contra	FR	0	+ + +	+	+ +
2	3 d	MCA/T	PE (AMI, VF), Stroke	79 / F	CAD, H, HF	Ipsi	O	–	+	+ +	+
						Contra	O	–	+ + +	+ +	+
3	3 d	MCA/T	Herniation of brain	72 / F	AS (EA), CAD	Ipsi	PR	3	0	+	+
						Contra	PR	0	+ + +	+	+ +
4	5 d 9 h	MCA/T	Stroke^*h^	48 / M	HC	Ipsi	FR	2	+	+	+
						Contra	FR	1	+ +	+	+ +
5	8.5 d	BA/T	Stroke	65 / F	CAD, H, HF	Ipsi	C	3	+	+	+ + +
						Contra	C	2	+ +	+	+
6	17 d	ICA/T, HT	PE, Stroke	75 / F	AF, AS, CAD, DM, H	Ipsi	FR	3	+	+ + +	+ + +
						Contra	FR	0	+ +	+	+ +
7	18 d	MCA/TE, HT	PE, Stroke	79 / M	AF, CAD, H, HF	Ipsi	PR	4	0	+ + +	+ + +
						Contra	PR	0	+ + +	+ +	+ +

^a^Abbreviations for the occluded vessel: BA, basilar artery; ICA, internal carotid artery; MCA, middle cerebral artery; T, thrombosis; TE, thromboembolism

^b^HT, hemorrhagic transformation

^c^Abbreviations for the cause of death: AMI, acute myocardial infarction; PE, pulmonary embolism; VF, ventricular fibrillation

^*^Convulsions in agonal phase

^d^Abbreviations for risk factors: AF, atrial fibrillation; AS, generalized arteriosclerosis; CAD, coronary artery disease; DM, diabetes mellitus; EA, carotid endarterectomy; H, hypertension; HC, hypercholesterolemia; HF, heart failure

^e^Abrreviations for brain regions: C, cerebellum; FR, frontal region; O, occipital lobe; PR, parietal region; P, pons

^f^Neuroscore: 0, no changes; 1, largely normal morphology but scattered neurons with nuclear abnormalities (pyknosis, low nuclear-cytoplasmic contrast, smearing of nuclear border); 2, large proportion of neurons with nuclear abnormalities; 3, large proportion of neurons with nuclear abnormalities while scattered neurons show signs of irreversible damage (shrunken cytoplasm with irregular borders and invisible nuclei); 4, large proportion of neurons show irreversible changes

^g^Grading of immunoreactivity: 0, no immunoreactivity; + , minimal immunoreactivity; +  + , moderate immunoreactivity; +  +  + , strong immunoreactivity

^h^Contralateral MCA occlusion 7 months previously

### Ischemic stroke induces delayed MANF protein expression in rat brain microglia/macrophages

**Fig. 2:**
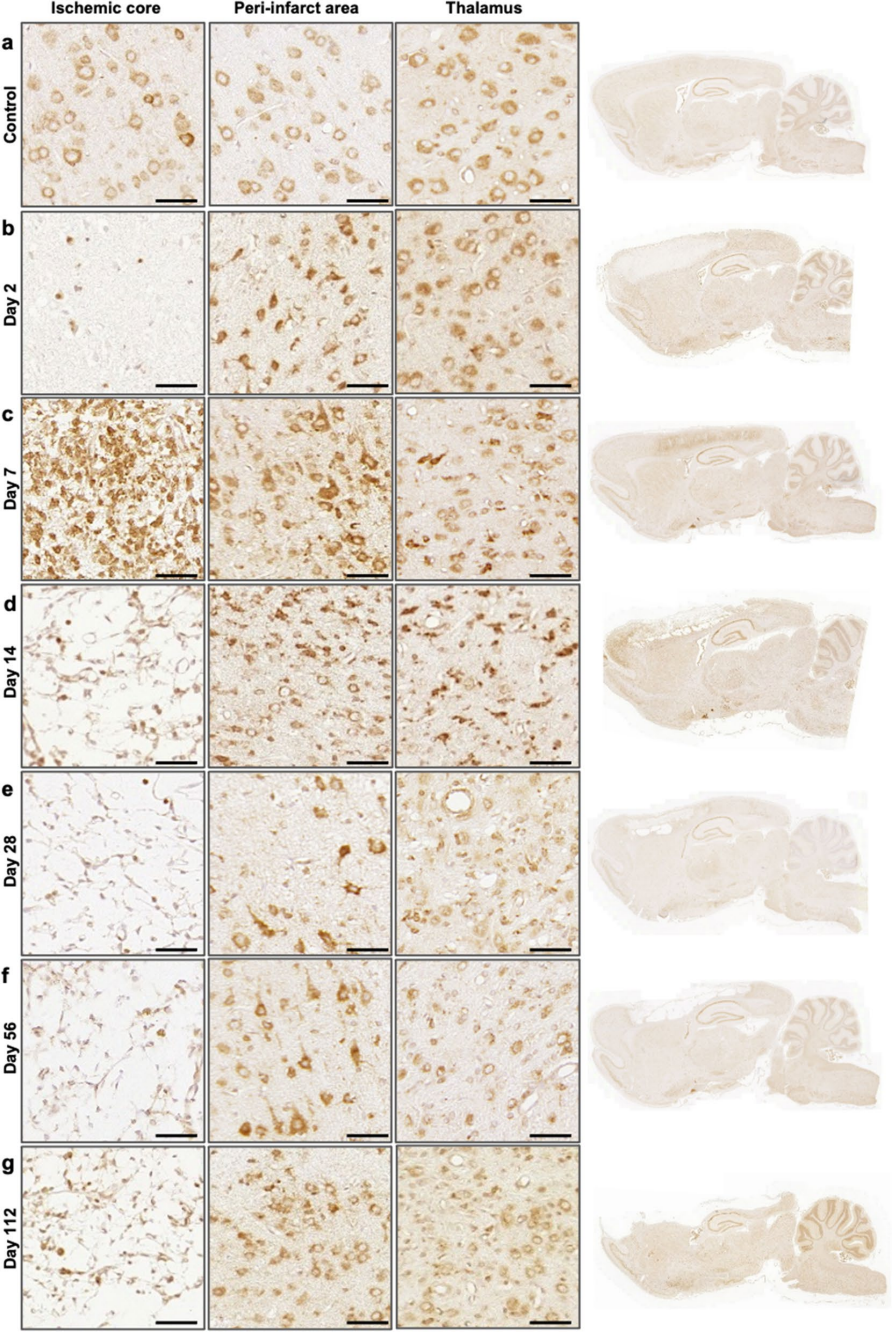
Temporal and spatial MANF protein expression in the rat brain post-stroke. Representative images of anti-MANF immunostaining from ischemic core in the cortex, peri-infarct area, thalamus, and the whole brain sagittal sections at 2 (**b**), 7 (**c**), 14 (**d**), 28 (**e**), 56 (**f**), and 112 (**g**) days after 90-min dMCAO in rat. Control images (**a**) are from the contralateral hemisphere of the stroke brain. Scale bar is 50 µm

To evaluate whether MANF expression post-stroke is similar in rat as we have found in the human brain, we next characterized MANF protein immunostaining patterns in a rat model of dMCAo at 2, 7, 14, 28, 56, and 112 days post-stroke (n = 4 per time point). Using rat model allowed us to investigate the MANF temporal expression profile in more detail as well as to use double immunofluorescence technique and identify MANF-positive cell phenotypes. In the non-stroke control brain MANF immunoreactivity was neuronal (Fig. 2a), as we have seen and reported before. In comparison to the contralateral hemisphere (Fig. 2a), MANF immunostaining was increased in the peri-infarct area at all time points until post-stroke day 112 (Fig. 2b–g). MANF expression was markedly decreased in the infarct core at day 2 (Fig. 2b and Additional file 1: Fig. 1) but increased in the corpus callosum (Additional file 1: Fig. 1). However, MANF immunostaining strongly increased in the infarct core on day 7 (Fig. 2c) and in the thalamus at day 14 (Fig. 2d). Temporally the increase in MANF immunostaining evolved similarly to the expression of the phagocytic marker CD68, which we have previously shown to peak in the same set of samples at day 7 in the ischemic core and at day 14–28 in the thalamus [5].

**Fig. 3:**
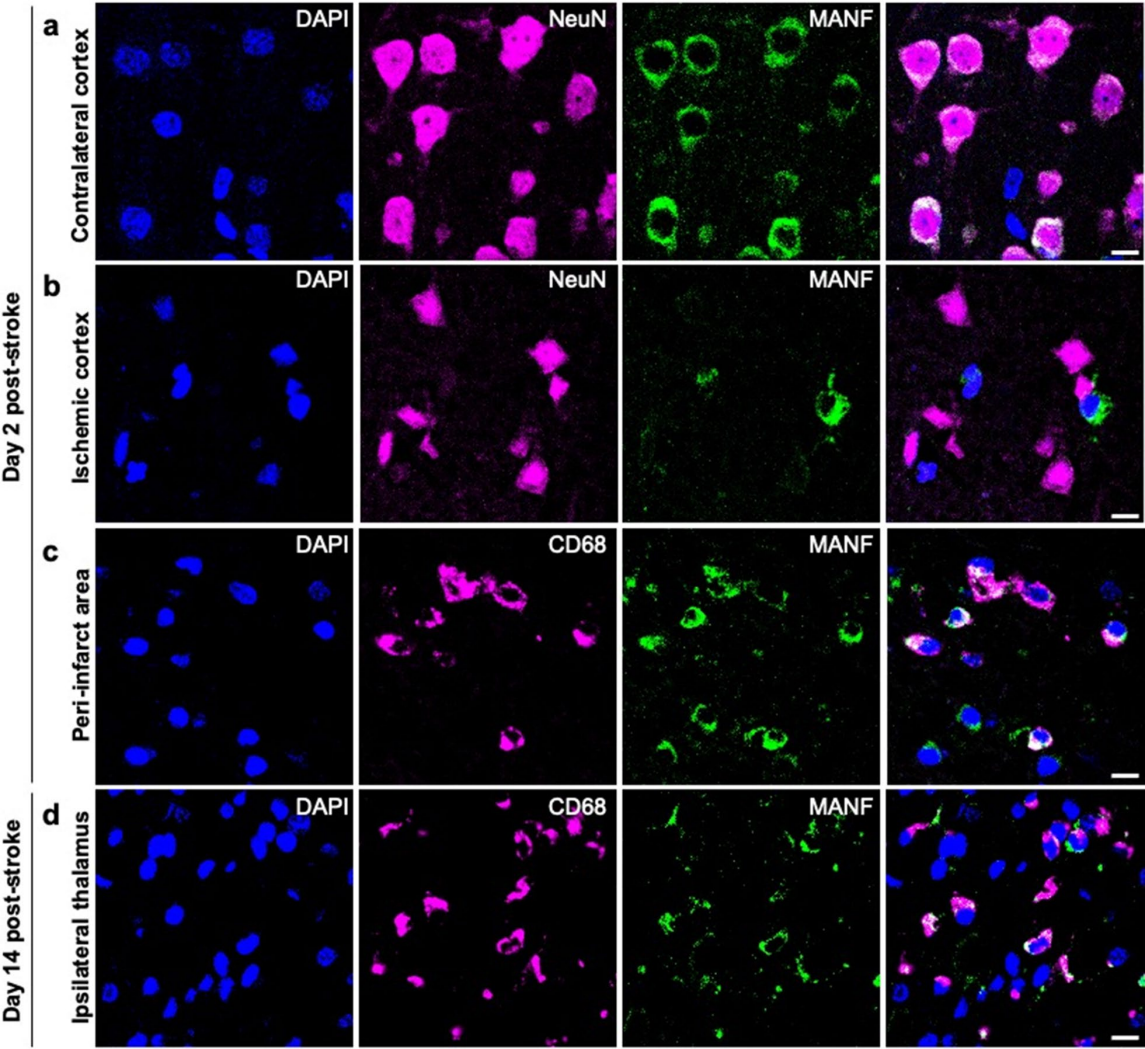
MANF is expressed in CD68 + cells in the ischemic cortex and ipsilateral thalamus. Double immunofluorescence staining of rat brain paraffin sections 2 (**a, b, c**) and 14 (**d**) days after 90-min dMCAo. NeuN and MANF colocalize in the contralateral (**a**) but not in the ischemic cortex (**b**). CD68 and MANF colocalize in the peri-infarct area (**c**) and in the ipsilateral thalamus (**d**). Scale bar is 10 µm

To identify which cells express MANF protein after stroke, we used double immunofluorescence staining on sections from the post-stroke rat brain. MANF and CD68 positive signals were found to be co-localized in the same cells in the post-stroke rat brain verified by confocal microscopy. In the contralateral cortex MANF co-localized with NeuN at every time point (Fig. 3a) but at day 2 in the injured ischemic cortex NeuN positive cells had lost MANF expression entirely (Fig. 3b). Only few MANF positive microglial cells were observed in the contralateral hemisphere. Notably, on day 2 and day 14 MANF co-localized with CD68 in the ischemic cortex and in the ipsilateral thalamus (Fig. 3c, d), representing MANF positive cells of the monocyte/macrophage lineage. We also found sparsely located GFAP positive astrocytes expressing MANF in the ischemic hemisphere but not in the contralateral brain (data not shown).

### MANF protein expression is induced in *Nestin*^*Cre/*+^*::Manf*^*fl/fl*^ knockout mouse brain after ischemic stroke

**Fig. 4:**
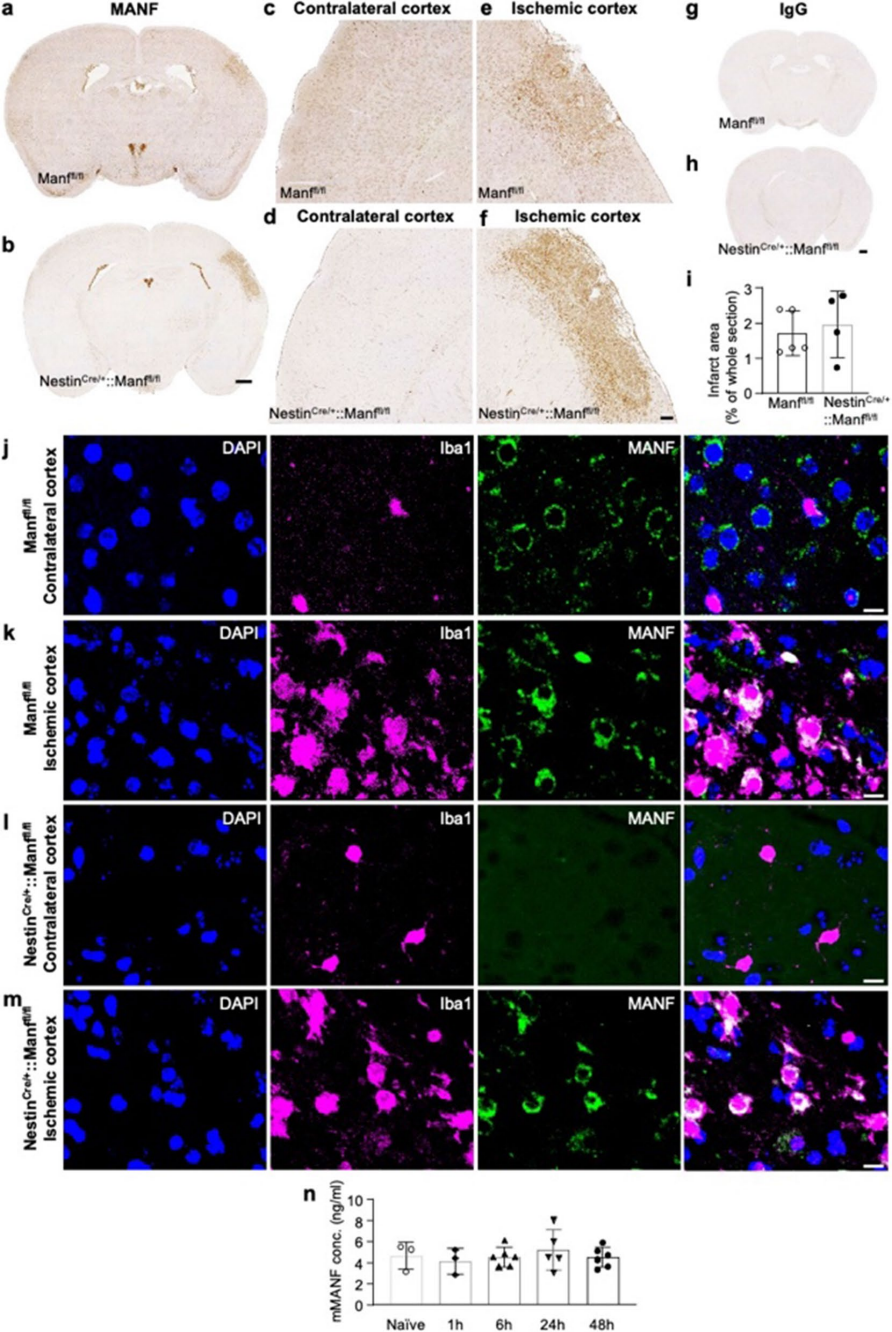
MANF protein expression is induced in Iba1 + cells of the Nestin^*Cre*/+^::Manf^*fl/fl*^ knockout mouse brain after ischemic stroke. Anti-MANF immunostaining of Manf^*fl/fl*^ control **(a, c, e)** and Nestin^*Cre*/+^::Manf^*fl/fl*^ knockout **(b, d, f)** mouse brain at 14d after permanent dMCAo. **g-h** Anti-rabbit IgG immunostaining of the same brain as in a, b to show the specificity of the rabbit anti-MANF antibody. **i** Average infarct area at 14d after permanent dMCAo in Manf^*fl/fl*^ wild type and Nestin^*Cre*/+^::Manf^*fl/fl*^ knockout mouse. a, b, g, h: scale bar is 500 µm. c-f: scale bar is 100 µm. **j-m** Anti-MANF and anti-Iba1 double immunofluorescence staining of the peri-infarct cortex of Manf^*fl/fl*^ control (**j**, **k**) and Nestin^*Cre*/+^::Manf^*fl/fl*^ knockout (**l**, **m**) mice at 14d after permanent dMCAo. Scale bar is 10 µm. **n** Endogenous MANF serum levels are not altered after permanent cerebral ischemia. Endogenous MANF levels were measured from wild type mouse serum with mouse MANF ELISA at different time points after permanent dMCAo, n = 3–6 per group. The values are expressed as mean ± SD. dMCAo = distal middle cerebral artery occlusion

Next, we used conditional MANF knockout mice where MANF is deleted from neurons and glial cells. We used genetically modified *Nestin*^*Cre/*+^*::Manf*^*fl/fl*^ mice with neuronal stem cell, neuronal, astroglial, oligodendrocytic, and oligodendrocyte precursor cellular *Manf* deletion to analyze post-stroke MANF expression using permanent dMCAo. In this mouse line, *Manf* is embryonically deleted from nestin-expressing cells by Cre recombinase that binds to loxP sites and deletes the *Manf* exon 3 leading to non-functional *Manf* gene [38, 68]. Microglia do not origin from nestin-expressing progenitors [29] and microglial *Manf* is therefore unaffected by Cre recombinase in the *Nestin*^*Cre/*+^*::Manf*^*fl/fl*^ mice. In the contralateral cortex of *Nestin*^*Cre/*+^*::Manf*^*fl/fl*^ mice MANF expression was absent (Fig. 4b, d, l) whereas in the *Manf*^*fl/fl*^ mice MANF was widely expressed in neurons (Fig. 4a, c). Interestingly, in the *Nestin*^*Cre/*+^*::Manf*^*fl/fl*^ mice MANF expression was induced in the infarct core, peri-infarct region, and the ipsilateral thalamus on day 14 (Fig. 4b, 4f) as was in the control mice (Fig. 4a, e). MANF in the *Nestin*^*Cre/*+^*::Manf*^*fl/fl*^ mouse ischemic cortex was shown to co-localize with the microglia/macrophage marker Iba1 (Fig. 4m), which confirmed that the post-ischemic MANF upregulation in the knockout mice was neither neuronal nor astroglial. At day 14, there was no difference in the infarct volume between the *Nestin*^*Cre/*+^*::Manf*^*fl/fl*^ and *Manf*^*fl/fl*^ mice (Student’s *t*-test *p* = 0.64, Fig. 4i).

### Endogenous MANF serum levels are not altered after permanent cerebral ischemia

To evaluate the potential of MANF as biomarker for stroke, we analyzed MANF levels from mouse serum post-stroke. Since cerebral MANF protein levels are changed after ischemia, MANF secretion is known to be induced upon ER Ca^2+^ depletion [21, 26], and increased serum MANF protein levels have been reported in Parkinson’s disease patients and newly diagnosed diabetic patients [18, 19, 73], we studied whether circulating MANF in serum could be used as a biomarker for ischemic stroke. We measured endogenous MANF levels from wild type mouse serum at 1 h, 6 h, 24 h and 48 h after permanent dMCAo. No significant differences in free circulating MANF levels after dMCAo were found when comparing to naïve mice without any ischemic damage (one-way ANOVA *p* = 0.80, Fig. 4n). The average concentration of mouse MANF was 4.6 ng/ml in serum.

### Intranasally delivered rhMANF reduces infarct volume and promotes recovery in a rat cortical stroke model

**Fig. 5:**
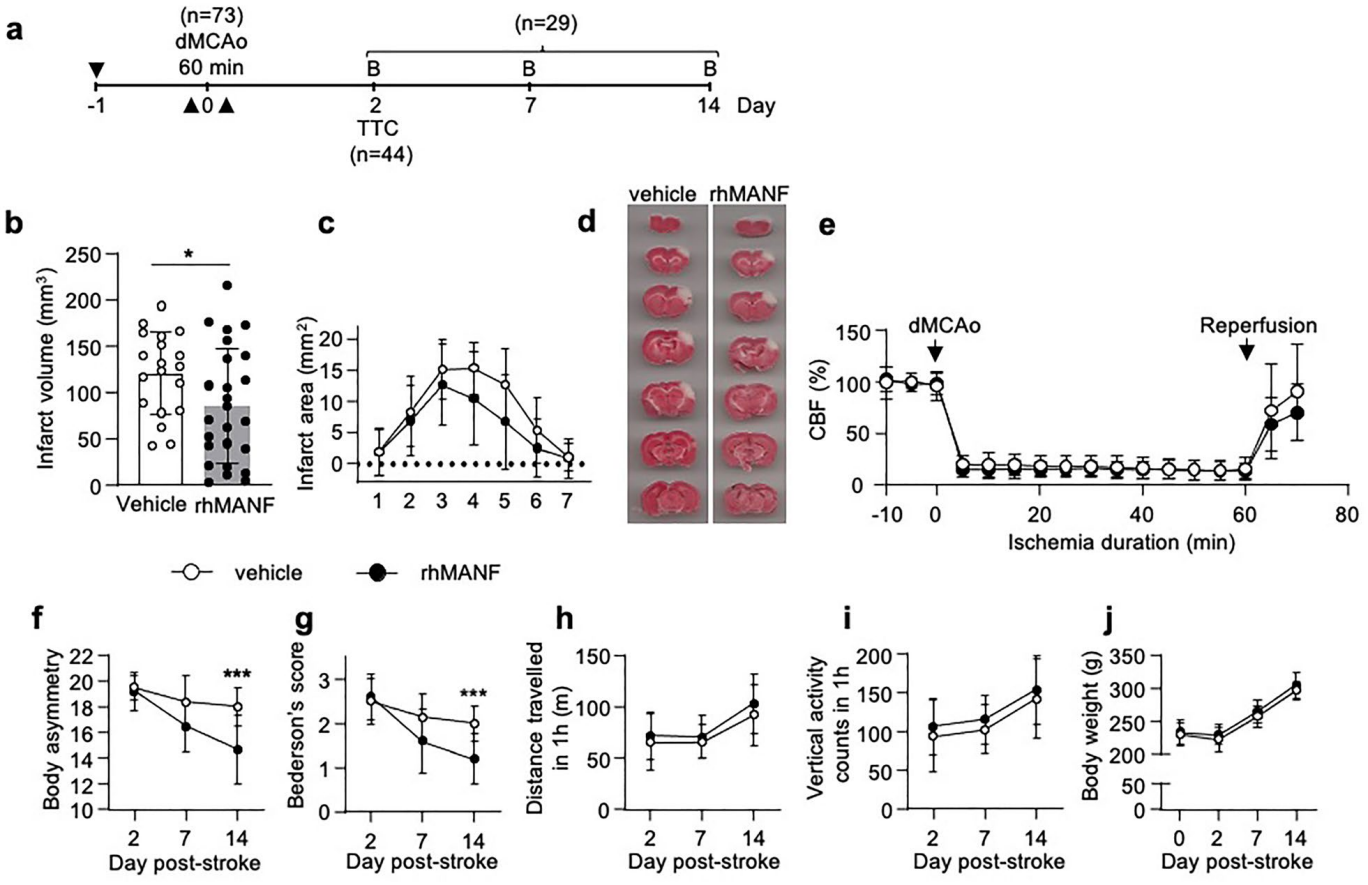
Pretreatment with intranasally delivered recombinant human MANF decreases infarct volume and promotes behavioral recovery after stroke. **a** Timeline of the experiment. The black arrows indicate the intranasal administration of rhMANF or vehicle. RhMANF or PBS was administered to the rats 3 times: 12 h before a 60-min dMCAo, immediately before dMCAo and immediately after reperfusion. The total MANF dose was 20 µg or 60 µg. dMCAo = distal middle cerebral artery occlusion, B = behavioral assay, TTC = 2,3,5-triphenyltetrazolium chloride staining. **b** Infarction volume was determined 2 days after dMCAo by TTC staining, n = 19–25 per group, (*p < 0.05), Student’s *t*-test. **c** Distribution of infarction along the rostrocaudal axis, n = 19–25 per group. **d** Representative images of TTC stained brain sections from the vehicle and rhMANF groups. **e** RhMANF treatment had no effect on cortical cerebral blood flow (CBF). CBF was measured with laser Doppler flowmetry before ischemia, during ischemia (dMCAo) and after reperfusion. Rats received intranasal rhMANF (n = 10) or vehicle (n = 9) immediately before CBF measurement. **f** Body asymmetry test, **g** Bederson’s score, **h** horizontal activity, **i** vertical activity, and **j** body weight at different time points after 60-min dMCAo in vehicle (n = 14) and rhMANF (n = 15) treated rats. The total MANF dose was 20 µg. In **f**–**g** (***p < 0.001) indicate comparison between vehicle and rhMANF groups with Mann–Whitney U test, corrected for multiple comparisons. The values are expressed as mean ± SD

As the intracranial delivery of MANF has been shown to be neuroprotective in ischemic stroke models, we conducted a proof-of-concept study using non-invasive intranasal delivery of rhMANF. Although, treatment before the dMCAo is not clinically relevant, it is relevant to explore whether MANF is neuroprotective with clinically relevant administration route. Each rat received three intranasal doses of recombinant MANF protein: 12 h before dMCAo, immediately before dMCAo, and immediately after reperfusion (Fig. 5a). Intranasal rhMANF pretreatment significantly reduced the infarct volume at day 2 (Student’s *t*-test *p* = 0.038, Fig. 5b–d). The rhMANF-treated rats had 30% smaller lesions than the vehicle-treated rats. No differences were found between the treatment groups in cerebral blood flow during dMCAo or reperfusion measured with Laser Doppler flowmetry (Fig. 5e).

In a second experiment, we explored whether the cytoprotective effect of intranasal rhMANF is associated with hastened recovery post-stroke. We monitored the recovery of the rats for 14 days after dMCAo. On day 14 post-stroke, there was a significant difference between the treatment groups in the body asymmetry test (Mann–Whitney U test *p* = 0.0004, Fig. 5f) and in the Bederson’s score (Mann–Whitney U test *p* = 0.0003, Fig. 5g). For day 7 post-stroke, the multiple comparisons corrected *p* values for the body asymmetry test and Bederson’s score were 0.051 and 0.058, respectively. There were no differences in the horizontal distance traveled, vertical activity or body weight between the treatment groups (Fig. 5h–j).

Next, we clarified the pharmacokinetic profile of MANF after intranasal administration. Evaluating the pharmacokinetic profile for potential and experimental drugs is essential for brain drug delivery and development because it allows exploring effectivity and safety as well as optimizing dosages. To quantify the bioavailability of rhMANF after intranasal delivery, we measured rhMANF levels in the brain and blood using ^125^I-labeled rhMANF and hMANF ELISA (Additional file 1: Fig. 2). In the dMCAo rats, approximately 0.4% (222 CPM/g) of the radioactivity of ^125^I-labeled rhMANF was found in the blood 60 min after intranasal administration (Additional file 1: Fig. 2b). Only 0.003% (17 CPM/g) of the radioactivity was detected in the brain and 0.08% (63 CPM/g) in the liver (Additional file 1: Fig. 2b). The average concentration of hMANF in the serum measured with ELISA was 850 pg/ml 60 min after intranasal administration of rhMANF, resulting in approximately 0.05% of the total rhMANF dose in the serum (Additional file 1: Fig. 2c). The levels of rhMANF in the brain homogenates were under the detection limit of ELISA i.e. below 45 pg/ml [18]. There was a trend towards a positive correlation between ^125^I-rhMANF levels in the brain and liver (Pearson correlation R = 0.793, *p* = 0.060). Also, the brain levels of ^125^I-rhMANF evolved in parallel, albeit not statistically significant in correlation, with the rhMANF serum levels detected with ELISA (Pearson correlation R = 0.742, *p* = 0.092).

### Intravenously administered rhMANF reduces infarct volume in a rat cortical stroke model and alters post-stroke cytokine levels in the brain and blood

**Fig. 6:**
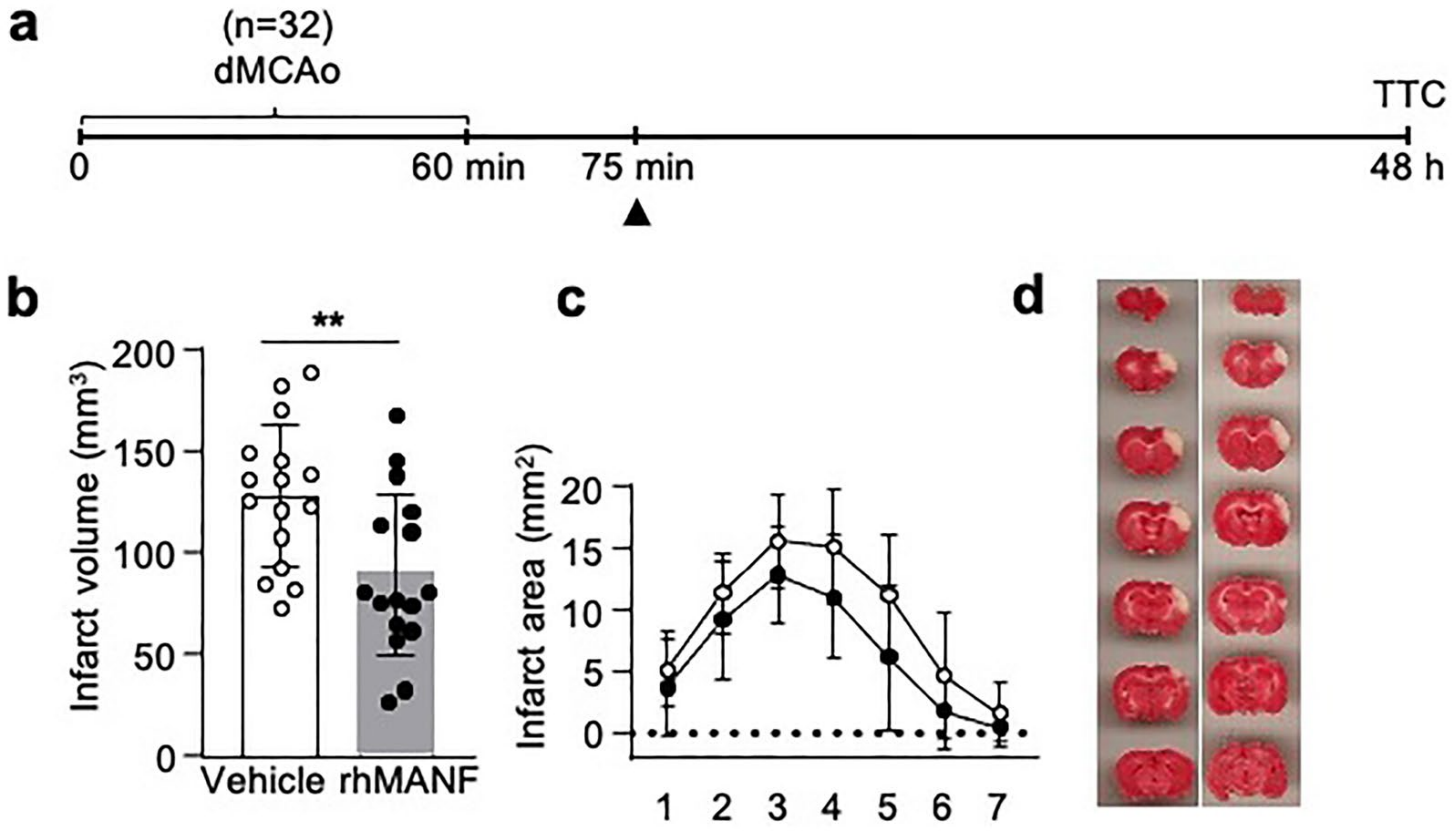
Post-reperfusion treatment with intravenous recombinant human MANF decreases infarct volume. **a** Timeline of the experiment. The black arrow indicates intravenous administration of rhMANF or vehicle. RhMANF (1.5 µg or saline was administered to the rats 15 min after reperfusion. dMCAo = distal middle cerebral artery occlusion, TTC = 2,3,5-triphenyltetrazolium chloride staining. **b** Infarction volume was determined 2 days after dMCAo by TTC staining, n = 16 per group, (**p < 0.01), Student’s *t*-test. **c** Distribution of infarction along the rostrocaudal axis.**d** Representative images of TTC stained brain sections from vehicle and rhMANF groups. The values are expressed as mean ± SD

As most rhMANF after intranasal administration was detected in the blood, we hypothesized that intravenous delivery of rhMANF would have a similar protective effect as with intranasal delivery. We chose a relatively low rhMANF dose as the rhMANF level measured with hMANF ELISA was about 0.85 ng/ml after intranasal delivery. First, a single 1.5 µg bolus of rhMANF was injected i.v. into the tail vein of the rats approximately 15 min after the dMCAo reperfusion and we quantified infarction volume 2 days after dMCAo using TTC staining (Fig. 6a). The i.v. therapy with rhMANF significantly reduced the infarction volume (Student’s *t*-test *p* = 0.006, Fig. 6b–d). It should be noted that there were relative low number of rats in this study as well as according to the Stroke Therapy Academic Industry Roundtable (STAIR) recommendations [17, 47, 59] we should use both genders of animals, aged animals as well as carry out the experiment in multi-center manner. Thus, these results show a clear neuroprotective effect and more studies are needed to clarify the translational potential.

We measured the bioavailability of rhMANF after i.v. administration from serum and cortex using hMANF ELISA [18]. In line with our preceding experiment with intranasal rhMANF delivery, a very minor amount of rhMANF can penetrate into the brain after ischemic stroke. Therefore, we chose to use a high 75 µg rhMANF dose to be able to detect it in the brain. A dMCAo was performed and one dose of vehicle or rhMANF was administered i.v. after the dMCAo reperfusion (Additional file 1: Fig. 3a). Blood samples were collected at 30 min and 60 min, and after transcardial perfusion with saline, the brains were collected. RhMANF was detected in the infarcted cortex 60 min after i.v. administration (Additional file 1: Fig. 3b), indicating that rhMANF can penetrate into the cerebral tissue, possibly due to infarct-induced BBB disruption that has been reported to occur already 25 min after transient MCAo [1, 64]. However, the amount of rhMANF in the infarct cortex was relatively small (average 32 pg/mg of total protein). RhMANF was not detected in the contralateral cortex in any of the samples (Additional file 1: Fig. 3b). The concentration of rhMANF in the serum was measured 30 min and 60 min after i.v. rhMANF (75 µg) administration. RhMANF levels in the serum were significantly reduced from 30 min (152 ng/ml; representing 1.9% of the total rhMANF dose injected) to 60 min (17 ng/ml, representing 0.22% of the total rhMANF dose injected) time-point and based on these data, the calculated half-life of rhMANF in the serum is about 10 min (Additional file 1: Fig. 3c).

**Fig. 7:**
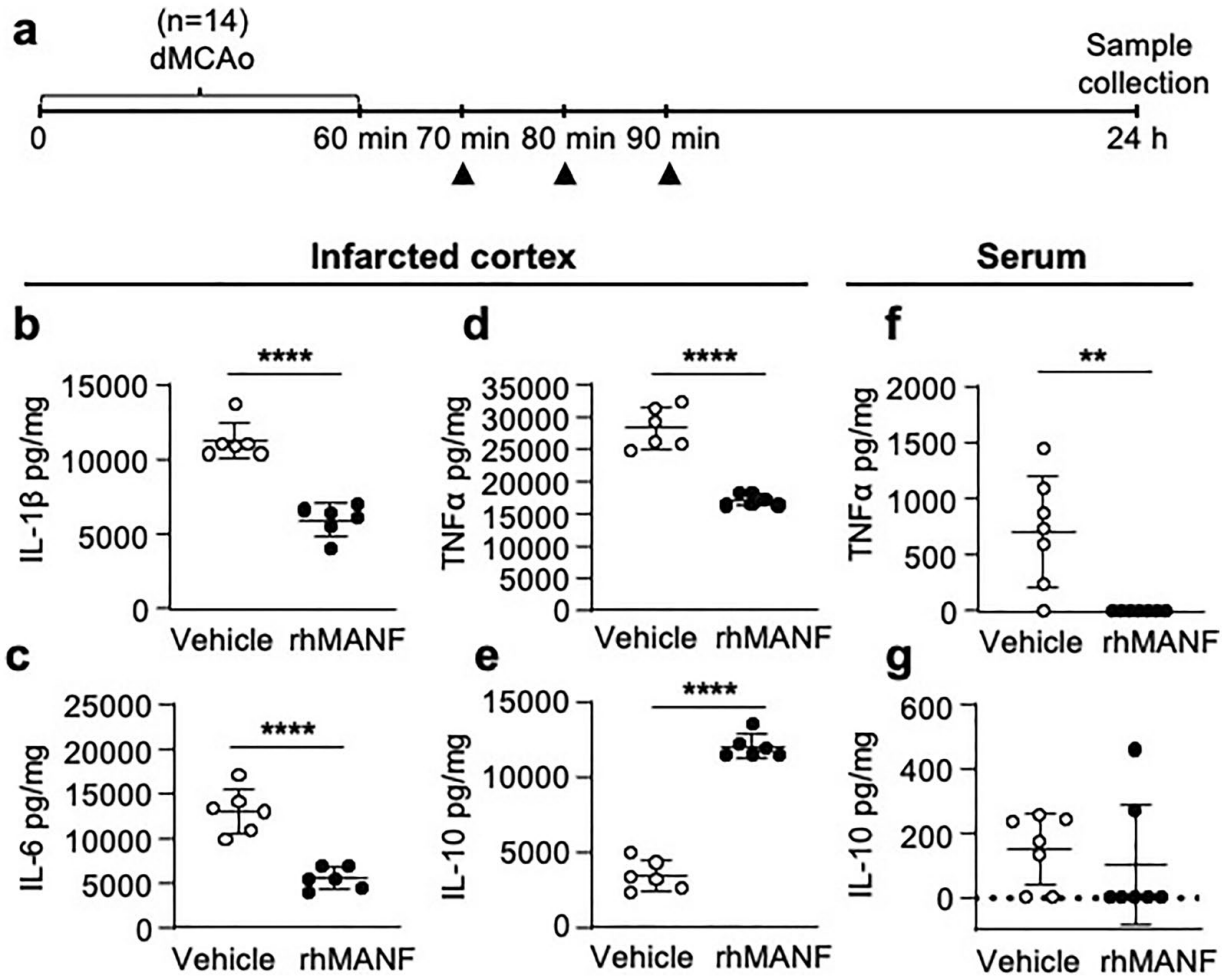
Intravenous administration of recombinant human MANF alters cytokine levels in the brain and blood after ischemic stroke. **a** Timeline of the experiment. Vehicle (saline) or rhMANF (1.5 µg × 3) was administered intravenously (i.v.) to rats that had undergone a 60-min distal middle cerebral artery occlusion. Arrowheads point the time of i.v. administration. After 24 h, the infarcted cortical tissue and serum were collected. **b**–**e** Concentration of IL-1β, IL-6, IL-10, and TNFα in the infarcted cortex 24 h post-dMCAo, normalized to the sample protein concentration (n = 6). (****p < 0.0001), Student’s *t*-test. **f-g** Concentration of TNFα and IL-10 in serum 24 h post-dMCAo, normalized to the serum protein concentration (n = 7). (**p < 0.01), Mann–Whitney U test. The values are expressed as mean ± SD

Since the elimination of rhMANF from the blood was very rapid, we increased the i.v. rhMANF dose to three boluses given within 10 min intervals. We hypothesized that the therapeutic effect of i.v. rhMANF would be mediated systemically and not from the brain. Since MANF has been associated with immunomodulatory effects, we measured cytokine levels from the brain and blood 24 h after dMCAo and i.v. rhMANF therapy (Fig. 7a). RhMANF treatment significantly reduced the concentration of IL-1β, IL-6, and TNF-α (*p* < 0.0001; Student’s *t*-test, Fig. 7b-d) in the infarcted cortex and increased the concentration of IL-10 (*p* < 0.0001; Fig. 7e). In the serum, the concentration of TNF-α was reduced (*p* = 0.005, Mann–Whitney U test; Fig. 7f) while the concentration of IL-10 was not affected (Fig. 7g) after the i.v. rhMANF treatment when compared to the vehicle group.

In the same experimental setup, we measured blood gases and electrolytes to rule out that i.v. rhMANF therapy would affect physiological parameters (Additional file 1: Fig. 4a). There were no statistically significant differences in any of the parameters measured (Additional file 1: Fig. 4b–i). Mean arterial blood pressure and heart rate were measured in naïve animals receiving three increasing doses of i.v. rhMANF boluses (Additional file 1: Fig. 4j–l) but we found no difference between the vehicle and rhMANF groups. We also tested if i.v. MANF treatment would affect the leakage of the BBB since BBB breakdown is known to exacerbate ischemic injury [58]. We administered Evans blue dye i.v. 48 h after dMCAo (Additional file 1: Fig. 5a) but found no statistically significant difference between the groups in Evans blue extravasation into the infarct area (*p* = 0.09, Student’s *t*-test, Additional file 1: Fig. 5b), although there was a trend towards a decrease in the rhMANF group.

## Discussion

This is the first study to show that after stroke MANF protein expression is triggered in microglia/macrophages in the human brain parenchyma. Similar spatiotemporal changes in MANF protein expression are found in the ischemic human and rodent brain. During the first days after ischemic stroke, MANF protein expression was decreased in the infarct core in both patients and rodents. However, when microglia/macrophages are activated in the post-ischemic brain, MANF protein expression is intensely present in those cells. The post-stroke inflammatory response is more delayed in human patients than in rodents, and the number of microglia/macrophages in ischemic brain tissue has been shown to be highest around 2 weeks post-stroke in patients [42], whereas in rats the peak is already at day 7 [5]. We observed that MANF had a similar expression pattern than the phagocytic marker CD68 in both patients and experimental animals, thus making dMCAo a relevant model for studying the role of MANF in inflammation and ischemia. The most important finding of this study is that there is drastic change of MANF protein expression towards microglia/macrophages after stroke. Although, in non-injured brains, MANF mRNA levels are relatively high in all cell types, the immunoreactivity experiments with MANF antibodies show protein to be found primarily in neurons. However, after stroke, there is a drastic change in this, and brain microglia/macrophages express robustly MANF protein. We showed this with human stroke patients' *postmortem* brains, rat brains and mouse MANF knockout brains where MANF was deleted from the neuronal lineage of cells (neurons, astrocytes, oligodendrocyte precursors and oligodendrocytes). Moreover, to bridge the gap between the translation of our findings from rodent models to humans, we explored more how systemic administration of MANF can enhance the recovery process after stroke using rat cortical ischemia–reperfusion model and found that enhancement of recovery correlates with inflammatory biomarkers.

The observation that the cerebral response to acute focal injury is reflected in the whole cerebrum is not new. We observed CD68 positive cells also in the contralateral hemispheres, especially in the white matter of infarcted human brains. In the same samples, elevated cyclo-oxygenase 2 and tumor necrosis factor α immunoreactivity, and increased density of intercellular adhesion molecule 1-expressing microvessels were found in the contralateral hemisphere when comparing to non-infarcted control brains [42, 56, 57]. These data suggest that inflammatory changes occur also in the contralesional human brain after ischemic stroke. Grossly, the contralateral hemisphere is not always completely intact tissue since ischemic stroke can cause brain herniation also influencing the contralateral side. Also changes in electrical activity, cerebral blood flow, and metabolism are known to occur contralesionally after ischemic stroke possibly representing diaschitic or reparative effects after injury [4], and may include inflammatory events as well. CD68 positive cells have been found in the healthy brain as well, particularly in the white matter, and to increase with age [27, 28]. Therefore, it is also possible that the observed CD68 expression in the contralateral hemisphere of infarcted brains represents basal CD68 expression in the human brain.

*Manf* mRNA is highly expressed in mouse microglia in the healthy brain [82] but microglial MANF protein expression has been reported to be very low [60]. We observed some MANF positive microglial cells in the contralateral hemisphere of rat brains, but it seems that translation of MANF protein is strongly induced after ischemia in activated microglia/macrophages. There are several possibilities for why MANF translation could be induced in the reactive microglia/macrophages. MANF may be needed for the increased production and secretion of proteins such as cytokines, or for ER remodeling during the activation of microglia/macrophages. The increased protein production in the activated microglia/macrophages may induce unfolded protein response and subsequent MANF translation. Shen *et al*. showed that the ER chaperone GRP78 and MANF were both induced in activated microglia after ischemia [60]. They also showed that microglial MANF upregulation is not specific for ischemia as the ER stress inducer tunicamycin triggered MANF expression as well and caused microglial activation in primary cultures. Moreover, activated microglia/macrophages are highly migratory and MANF may be needed in the ER for enabling motility as ER needs to be flexible in motile cells. It has been shown that endogenous and exogenous MANF is important for neuronal migration [69, 70]. Since there is large demand to ER in phagocytic cells, we postulate that MANF protein expression in microglia/macrophages is induced because of the load that the changing morphology of the ER in phagocytic cells induces. Moreover, we have not been able to distinguish between microglia and macrophages, and whether there are differences in MANF protein expression in the two cell types. It is also possible that MANF immunoreactivity in microglia/macrophages after stroke is originating from circulating MANF, and in this case these cells would take it up and therefore become MANF-positive for immunoreactivity. We have previously shown that in *Manf*^*fl/fl*^ control mice and in *Nestin*^*Cre/+*^*::Manf*^*fl/fl*^ mice *Manf* mRNA expression is increased in the ischemic cortex after stroke [48]. In addition, we have previously shown that *Nestin*^Cre/+^*::Manf*^*fl/fl*^ mice have increased lesion volume 2 days after stroke [48], indicating that endogenous neuronal MANF is neuroprotective. Moreover, the microglia field is developing promptly, and new microglia phenotypes have been identified [62, 63], that remain to be studied in relation to MANF or stroke.

MANF may also be important for frank phagocytosis as it is expressed particularly in the round, most reactive state of microglia/macrophages, or MANF may be needed for immune cell recruitment. It has been shown that AAV-MANF increases the number of CD68 positive cells in the peri-infarct area 4 days after dMCAo [48] and that intravitreal rhMANF injection increases the number of CD11b positive cells in the damaged retina [51].

Additionally, MANF may be secreted from immune cells and could help to restore the homeostatic environment in the injured tissue. At least in vitro, endogenous MANF is known to be secreted from non-neuronal cells and the secretion is greatly enhanced upon ER Ca^2+^ depletion [7, 21, 65]. In vivo, MANF has been shown to modulate microglia/macrophage activity toward the regenerative M1 type in a paracrine manner [51, 77]. It would be highly interesting to generate a mouse line with microglia/macrophage specific MANF deletion and study whether the recovery from stroke is hindered in these mice compared to wild type.

Our understanding of the role of exogenous MANF and how it mediates its cytoprotective effects is still limited [45]. Neuroplastin has been suggested to function as a plasma membrane receptor for MANF with modest binding [75] and sulphatide-mediated cellular uptake has been postulated as the mechanism of how extracellular secreted/exogenous MANF enters cells [8]. Endogenous MANF seems to be important primarily in maintaining ER homeostasis [38, 52]. However, the role of exogenous MANF may be different, and exogenous MANF may interact with multiple target proteins intracellularly. Interestingly, the N-terminal RTDL amino acid sequence of MANF, which functions as an ER retention signal, is not required for the in vivo neuroprotective effect of recombinant MANF protein in cerebral ischemia [50], implying that the neuroprotective effect of exogenous MANF may not be directly related to ER homeostasis. Furthermore, increasing amount of data shows that MANF has immunomodulatory effects (recently reviewed in [45].)

We measured endogenous MANF levels from mouse serum at different time points during the first 2 days after ischemic stroke but found no difference compared to naïve animals. The level of endogenous MANF in serum was 4.6 ng/ml in mouse. In humans, serum MANF concentration has been reported to be between 3.5 and 6 ng/ml in healthy adults [18–20, 61, 71] and less, approximately 2.5 ng/ml, in the aged [61]. Increased serum MANF protein levels have been reported in patients with Parkinson’s disease diagnosed on average 6 years ago [19], indicating that circulating MANF levels may be altered in chronic CNS disease. However, we were interested in the potential of MANF in acute diagnostics of stroke, but our data do not support the use of free circulating MANF as a potential biomarker in ischemic stroke during the first 2 days after stroke. Furthermore, it is unclear from where the free circulating MANF originates. In human blood cells, MANF protein has been detected mostly in platelets, to some extent in leukocytes, and very little in red blood cells but at least in the case of Parkinson’s disease patients the increased serum MANF levels were not originating from the blood cells [19]. However, more relevant biomarker for stroke could be detection of cytokines from the serum. The amount of pro-inflammatory cytokines was downregulated, and anti-inflammatory cytokines upregulated in the infarcted cortex and serum 24 h after intravenous rhMANF treatment. Similar results were found by Han *et al*. [23] where intraventricular rhMANF therapy decreased pro-inflammatory cytokine levels in the infarction area after MCAo in aged mice. The anti-inflammatory effect of rhMANF was shown to be dependent on the TLR4/MyD88/NF-κB pathway in vitro [23]. A vast amount of data from different disease models has shown that MANF regulates the NF-κB pathway [45].

Intracranial delivery of MANF is neuroprotective but not feasible in ischemic stroke patients due to increased risk of hemorrhage caused by thrombolytic treatment and the infarct itself, and therefore it is vital to explore the possibility for non-invasive delivery. There is evidence that intranasally delivered peptides reach the central nervous system in humans [55]. As an attempt to develop a non-invasive method for administering MANF, we show for the first time that intranasally delivered rhMANF reduced infarction size and promoted post-stroke recovery in rats. However, only 0.003% of the ^125^I-rhMANF dose was detected in the brain 1 h after intranasal administration and would theoretically result in approximately 20 pM brain concentrations. Most rhMANF after intranasal administration was found in the blood. The calculated bioavailability of ^125^I-rhMANF in the blood (0.4%) was higher compared to unlabeled rhMANF in the serum (0.05%) which may suggest either counting of free radioactivity, binding of rhMANF to a carrier protein masking the epitopes for ELISA, or binding of rhMANF to blood cells that are removed during sera sample preparation. The low levels of ^125^I-rhMANF detected in the brain may also have originated from the systemic circulation after stroke-induced disruption of the BBB. It has been reported that in the intraluminal MCAo rat model there is a leakage of large molecules through the BBB already 25 min after reperfusion [1, 64]. Intranasal rhMANF therapy increased the MANF serum concentration about 0.85 ng/ml i.e., 20% compared to the endogenous level. However, interindividual variation in the rhMANF serum levels was high, likely reflecting differences in absorption in individual rats, which is a known problem in the intranasal delivery route [44]. The interindividual variation after intranasal rhMANF delivery is reflected in the stroke outcome as the variation in the infarction volume was high as well. It is possible that the neuroprotective effect of intranasal rhMANF was due to systemic effects or effects on the brain endothelium. As the concentrations in the brain and serum were low after intranasal MANF administration, it is likely that the neuroprotective effect is mediated by other than direct effect in the brain. It remains speculative since we do not know it and since the mechanism of action of exogenously added MANF still remains mostly elusive, it is difficult to speculate further. Therefore, we chose to administer rhMANF i.v. and found neuroprotective effects on lesion volume also after i.v. therapy. Surprisingly, interindividual variation in rhMANF serum levels was still high. Furthermore, the half-life of rhMANF in the serum was short, about 10 min, and significantly shorter than the half-life in the brain parenchyma, which is expected to be 5.5 h [determined for the MANF homolog, rhCDNF [49]. The fast clearance of rhMANF from blood circulation could reflect rapid proteolytic degradation, renal excretion, hepatic metabolism, tissue distribution, or binding to blood cells. Proteins smaller than 70 kDa are known to eliminate via renal clearance [81]. The amount of rhMANF in the infarcted cortex after intravenous administration was small (average 32 pg/mg of total protein) compared to the amount of endogenous MANF in the mouse brain [250 ng/mg of total protein [14]], only 0.01% of the endogenous amount, indicating that the brain levels acquired after systemic administration of rhMANF are not biologically significant. Therefore, we hypothesize that the therapeutic effect of rhMANF in ischemic stroke derives from the systemic circulation, possibly via modulation of immune cell phenotype. Additionally, in stroke and traumatic brain injury models, intracranial MANF treatment has been shown to decrease brain edema and BBB leakage [23, 34, 35, 74]. However, we found no statistically significant effect on the BBB integrity after i.v. rhMANF delivery in ischemic stroke.

## Conclusions

This study demonstrates the analogous spatiotemporal evolution of post-ischemic MANF expression in rat and human brain (Figs. 1, 2, 3 and 4). We demonstrate that there is a drastic change in MANF protein expression pattern after stroke in microglia/macrophages. MANF is evidently expressed in phagocytic microglia/macrophages especially around 2 weeks in patients and 1–2 weeks post-stroke in experimental animals. Our findings provide important insight into how endogenous MANF may contribute to post-stroke inflammation, supporting further investigation into MANF-based therapeutic applications. We also show that the intranasal administration of rhMANF was neuroprotective by restricting the infarct size and by promoting functional recovery. Furthermore, intravenous administration of rhMANF was neuroprotective and had anti-inflammatory effects. These results may have clinical implications as non-invasive administration delivery is needed for therapeutic proteins and show further evidence of the prominent anti-inflammatory effects of MANF in ischemic stroke.

### Supplementary Information

**Additional file 1**.

## Data Availability

Data will be given upon request.

## Abbreviations

BBB: Blood–brain barrier
CBF: Cerebral blood flow
CCA: Common carotid artery
dMACo: Distal middle cerebral artery occlusion
ER: Endoplasmic reticulum
LDF: Laser Doppler flowmetry
MANF: Mesencephalic astrocyte-derived neurotrophic factor
PBS: Phosphate-buffered saline
Rh: Recombinant human
