# Supplementary material for: MANF protein expression is upregulated in immune cells in the ischemic human brain and systemic recombinant MANF delivery in rat ischemic stroke model demonstrates anti-inflammatory effects
Source: Acta Neuropathol Commun. 2024 Jan 16;12:10. doi: 10.1186/s40478-023-01701-y (PMC10792833; doi:10.1186/s40478-023-01701-y)
Supplement: Supplementary file 1 — Additional file 1. [file 40478_2023_1701_MOESM1_ESM.docx]

**Supplementary Figures**


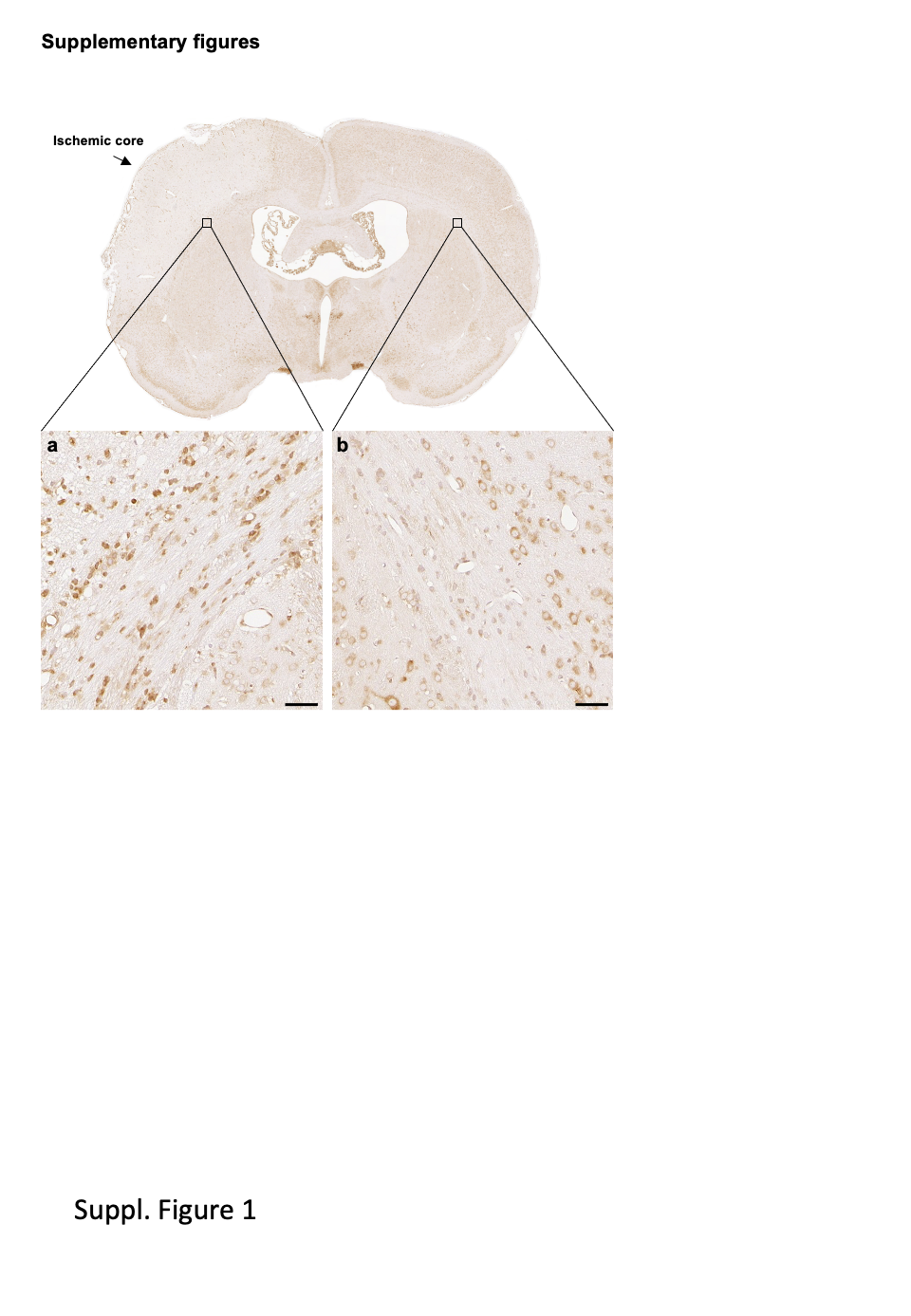


**Supplementary Fig. 1** MANF is downregulated in the ischemic core but upregulated in the ipsilateral corpus callosum at d2 post-stroke in rat. Representative image of anti-MANF immunostaining from ipsilateral (**a**) and contralateral (**b**) corpus callosum in coronal rat brain paraffin section 2 days after 90-min dMCAo. Scale bar is 50 µm.


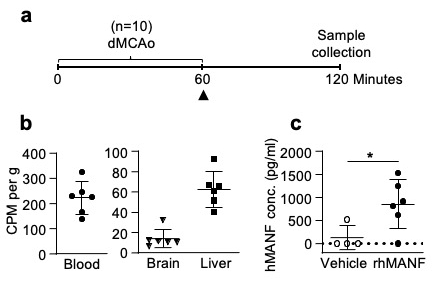


**Supplementary Fig. 2** Recombinant human MANF was detected in the systemic circulation, brain, and liver 1 h after intranasal administration. **a** Timeline of the experiment. Vehicle (PBS) or ^125^I-rhMANF (28 ng; 1081152 CPM) with unlabeled rhMANF (20 µg) was administered intranasally to rats that had undergone a 60-min distal middle cerebral artery occlusion. After 60 min, blood was collected, and the rats were perfused with saline. Liver and brain samples were collected after perfusion. **b** Radioactivity counts per minute (CPM) normalized to the sample weight (g) 60 min after intranasal administration of ^125^I-rhMANF (n=6) in the blood, brain, and liver. **c** RhMANF concentration in rat serum 60 min after intranasal administration of 20 µg rhMANF (n=6) or vehicle (n=4). * (p<0.05), Student’s t-test. The values are expressed as mean ± SD.


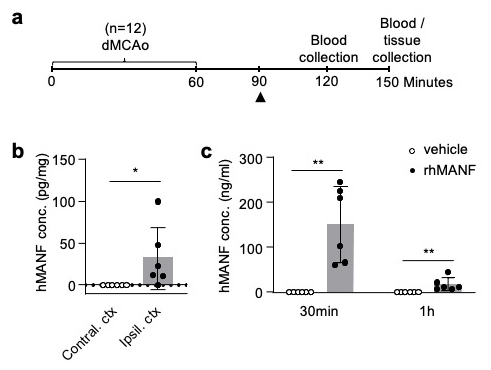


**Supplementary Fig. 3** Distribution of rhMANF after intravenous administration measured with hMANF ELISA. **a** Timeline of the experiment. Arrowhead points the time of i.v. administration. **b** Concentration of rhMANF (ng/mg total protein) in the contralateral and infarcted ipsilateral cortex 60 min after intravenous rhMANF (75 µg) administration. * (p<0.05), Mann-Whitney U test. **c** Concentration of rhMANF (ng/ml) in serum 30 min and 60 min after intravenous administration (75 µg) of rhMANF or vehicle. ** (p<0.01) Mann-Whitney U test, corrected for multiple comparisons. The data are expressed as mean ± SD.


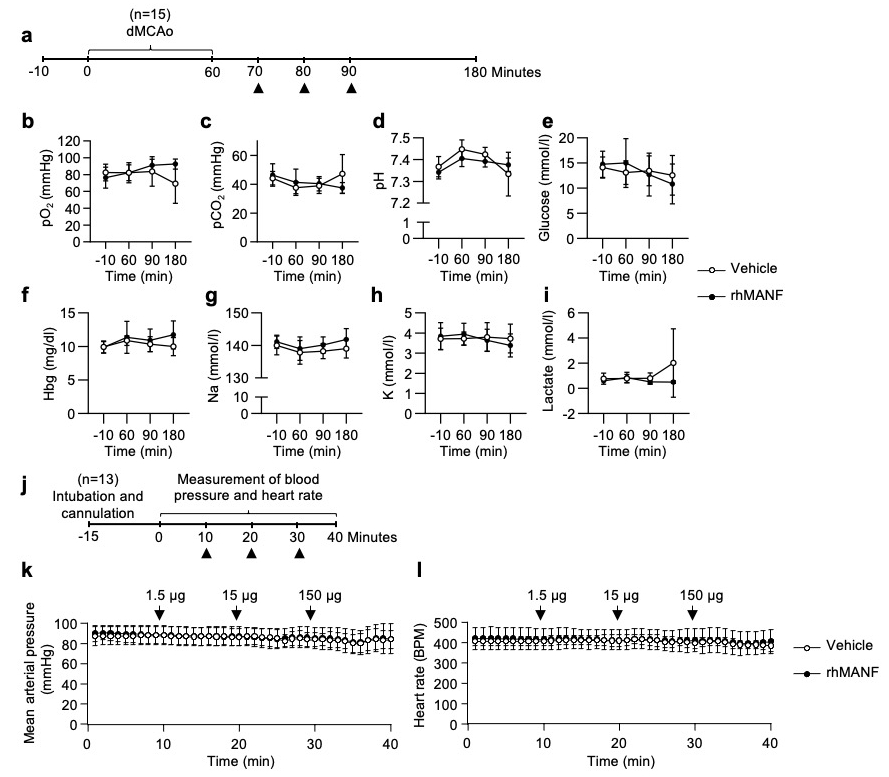


**Supplementary Fig. 4** Intravenous rhMANF therapy does not have influence on blood gasses, electrolytes, blood pressure, and heart rate. **a** Timeline of the experiment. **b-i** Arterial blood gasses and electrolytes were measured at different time points before and after 60-min distal middle cerebral artery occlusion, vehicle (n=7), rhMANF (n=8). **p<0.01, two-way ANOVA and Bonferroni’s *post hoc* test. **j** Timeline of the experiment. **k** Mean arterial blood pressure (mmHg) and **l** heart rate (beats per minute) were continuously measured from the carotid artery of naïve animals after administration of i.v. vehicle (n=6) or increasing i.v. rhMANF (n=7) boluses every 10 min. The data are expressed as mean ± SD.


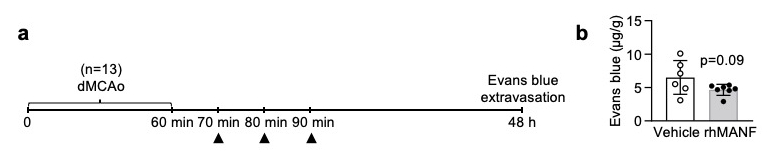


**Supplementary Fig. 5** Intravenous administration of recombinant human MANF after ischemic stroke has no effect on the Evans blue extravasation on the infarcted cortex. **a** Timeline of the experiment. Vehicle (saline) or rhMANF (1.5 µg x 3) was administered intravenously (i.v.) to rats that had undergone a 60-min distal middle cerebral artery occlusion. Arrowheads point the time of i.v. administration. After 48 h, the infarcted cortical tissue was collected. **b** Concentration of Evans blue dye (µg/g of tissue) in the infarcted cortex 48 h post-dMCAo (n=6-7). p=0.09, Student’s *t*-test. The values are expressed as mean ± SD.
